# Size and Beyond: A Comparative and Prospective Analysis on Asymmetric Flow Field-Flow Fractionation and Size-Exclusion Chromatography in Nanopharmaceutical Characterization

**DOI:** 10.1021/acs.analchem.6c01859

**Published:** 2026-09-17

**Authors:** Sara Tengattini, Caterina Temporini, Gabriella Massolini

**Affiliations:** Department of Drug Sciences, 19001 University of Pavia, via Taramelli 12, Pavia, Province of Pavia 27100, Italy

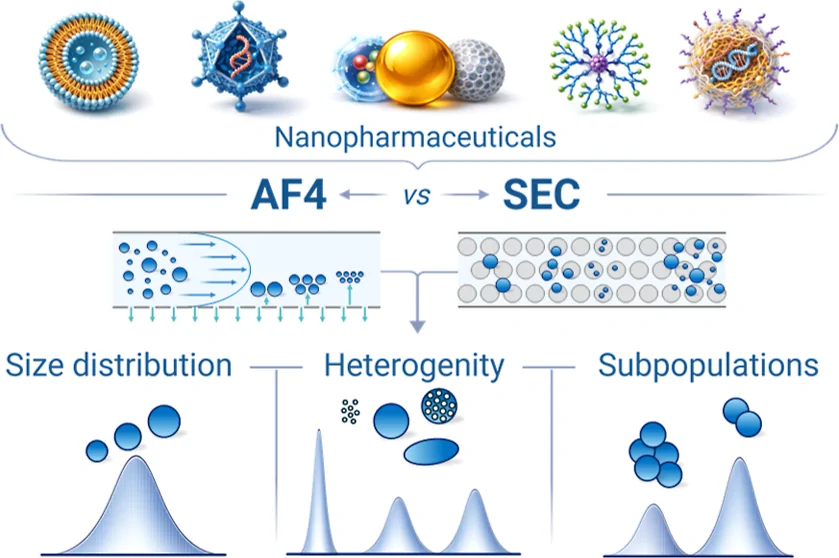

## Introduction

Nanopharmaceuticals (NPhs) are therapeutic products in which the active pharmaceutical ingredient (API) is delivered via a nanoscale delivery system. Although their dimensional range is generally defined as 1–100 nm, it can extend up to 1000 nm when the product still exhibits nanoenabled behavior, reflecting a functional rather than strictly size-based definition.^1^ These nanocarriers play a critical role in enhancing bioavailability, modifying the release rate, and targeting the drug to the required site, while also protecting the API against degradation.^2^ By combining the active molecule with a nanoscale carrier, NPhs can overcome limitations of conventional formulations and achieve improved therapeutic efficacy, addressing complex diseases and advancing personalized therapeutic strategies. A medicinal product can be designated as an NPh only if the nanoengineering is an integral part of the manufacturing process, and the nanomaterial employed is either indispensable for the therapeutic effect or provides distinct and additional functional properties to the active substance.^2^ Several nanoscale drug-delivery systems have been developed; the currently marketed ones include lipid-based carriers, polymeric nanoparticles (NPs), protein-based NPs, and inorganic NPs (INPs).^3^

The first NPh was approved in 1995 with the introduction of liposomal doxorubicin, marking the onset of clinical nanomedicines. Since then, the global NPh market has undergone significant growth. In recent market analyses, the global NPh market was estimated at $237.59 billion in 2024 and is projected to expand at a compound annual growth rate of 10.1%, reaching $562.93 billion by 2032.^4^ Although approved NPhs cover a wide range of therapeutic areas, oncology remains the predominant application, alongside a significant recent expansion into immune-related indications.^2,5^

The structural complexity of NPhs entails the definition and monitoring of a broad set of physicochemical properties that represent critical quality attributes (CQAs) in ensuring product safety and efficacy. Core CQAs include particle size and size distribution, directly affecting biodistribution, clearance, and interactions with biological barriers, as well as surface charge and chemistry, which influence stability and protein corona formation.^6^ Other key parameters are drug loading and encapsulation efficiency (EE), release kinetics, and stability under storage and physiological conditions. Depending on the nature of the nanocarrier, additional attributes must be considered, such as lipid composition for lipid-based NPs or polymer molecular weight (MW) distribution for polymeric systems.^7^

A wide range of analytical techniques is employed to address these attributes, including dynamic light scattering (DLS), NP tracking analysis (NTA), Taylor dispersion analysis (TDA), asymmetric flow field-flow fractionation (AF4), size-exclusion chromatography (SEC), and hydrodynamic chromatography (HDC) for size and size heterogeneity characterization; electrophoretic light scattering and capillary electrophoresis (CE) for electrophoretic mobility and surface charge; cryo-transmission electron microscopy (cryo-TEM) for morphology; and liquid chromatography–mass spectrometry (LC–MS) for chemical composition. Orthogonal methods are strongly recommended to build a comprehensive characterization package to be used during formulation development and for manufacturing quality control purposes. Regulatory agencies such as the European Medicines Agency (EMA) and the US Food and Drug Administration (FDA) have emphasized the need for such detailed analysis in their guidelines on nanomedicines,^1,8^ aligning with the ICH Q8 framework, where the systematic identification of CQAs is central to pharmaceutical development.

As the structural complexity of nanocarriers becomes increasingly evident and the pharmacological and safety implications of heterogeneity, aggregation, and degradation are progressively recognized, it has become clear that analytical strategies must move beyond simple average size determination toward advanced methods capable of generating size-resolved profiles of NPhs. In this context and in light of the growing regulatory emphasis on understanding NPh heterogeneity and stability, a critical evaluation of separative approaches capable of fractionating NPs based on size appears particularly timely and relevant. Accordingly, rather than providing a broad and superficial overview of all analytical techniques employed in NPh characterization, this review aims to deliver an in-depth and critical comparison of two of the most prominent size-based separation techniques currently applied in the field: AF4 and SEC. HDC has also been proposed as a size-based separation technique for NP analysis, with most applications focusing on polymeric and INPs,^9–13^ and only a few studies reporting its application to lipid-based systems.^14,15^ However, these examples remain limited and are not representative of the broad and complex landscape of fragile NPhs. For these reasons, HDC has not been included in the present comparative study.

The deliberate focus on AF4 and SEC enables a detailed assessment of their respective strengths, limitations, and potential evolution based on a critical review of applications reported since 2020. In addition, the regulatory context is considered to outline emerging challenges and future perspectives for size-based separation techniques in this field.

### Asymmetric Flow Field-Flow Fractionation and Size-Exclusion Chromatography: Principles and Detectors

AF4 and SEC are elution-based separation techniques commonly used for the separation and characterization of macromolecules. Both techniques provide size-based separation related to the hydrodynamic properties of analytes in solution, although they do so through fundamentally different mechanisms.

The following sections provide only a brief overview of AF4 and SEC separation mechanisms, since this review is not intended to address their theoretical principles. Comprehensive reviews can be found elsewhere for both techniques.^16–23^

### Asymmetric Flow Field-Flow Fractionation

AF4, the most widely used among FFF techniques, is a liquid, one-phase, chromatography-like separation technique that operates without a stationary phase. It is based on the differential transport of analytes in a thin, ribbon-like channel. The channel consists of two plates, with the lower plate covered by a semipermeable membrane. A laminar liquid flow moves the sample through the channel, while a perpendicular crossflow is applied toward the membrane. Macromolecules and particles are driven toward the membrane by this crossflow, but their position is counterbalanced by diffusion. Small species diffuse further away from the membrane, reaching faster streamlines of the parabolic flow profile, while larger species remain closer to the membrane in slower streamlines. As a result, analytes are separated according to their diffusion coefficient, which is related to their hydrodynamic size via the Stokes–Einstein equation, leading to the sequential elution of analytes, with the smallest species detected first (Figure 1A).

**Figure 1:**
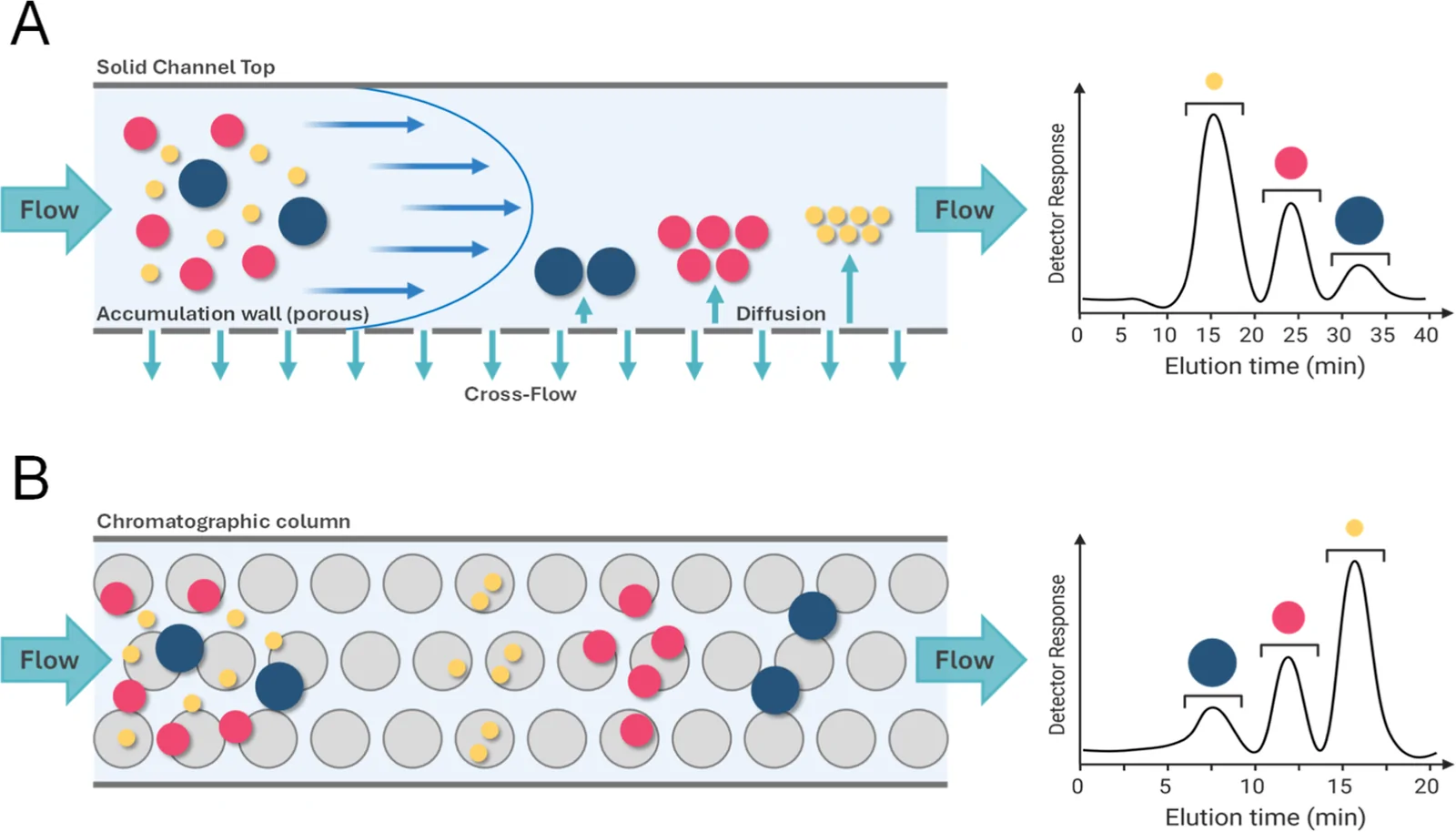
Schematic representation of the separation mechanism in (A) AF4 and (B) SEC.

In the past two decades, AF4 has rapidly evolved into a powerful analytical separation technique for the characterization of macromolecules and nanoscale particles. Today, it is widely established as a standard method for quantifying protein aggregation, determining size and molar mass of ultralarge biopolymers, as well as for analyzing particles.^19,23–27^

### Size-Exclusion Chromatography

SEC is a liquid chromatographic technique that separates analytes by exploiting their differential access to the pores of the stationary phase. The stationary phase is composed of porous particles, and the pore size determines the volume accessible to molecules with different dimensions. Large species, with hydrodynamic radii exceeding the pore size, are completely excluded from the stationary phase and, thus, elute first. Molecules of intermediate size partially penetrate the pores and elute later, whereas the smallest molecules can access the entire pore network, resulting in the longest retention times (Figure 1B). Since, in the ideal case, the separation mechanism is purely entropic and does not involve chemical interactions, SEC can be performed under mild, near-physiological conditions.

In the pharmaceutical field, SEC plays a pivotal role in the characterization of therapeutic proteins, where it is the reference method for detecting and quantifying size variants that represent a CQA. It is also widely used for the analysis of polymers and, more recently, has seen increasing application in the study of NPs.^20^

### Online Detection Options and Multi-Detector Strategies

Both SEC and AF4 can be coupled to a wide range of detectors, providing different pieces of information.

The most common options for concentration detectors include ultraviolet (UV), differential refractive index (dRI), and fluorescence detectors (FLD) depending on the nature of the analyte. The dRI detector provides a near-universal response and is particularly suitable for NPs and nonchromophoric polymers, although with generally lower sensitivity than optical methods. UV–vis detection offers greater sensitivity and selectivity for species bearing chromophores. FLD further enhances sensitivity and specificity when the analyte is intrinsically fluorescent or can be fluorescently labeled.^28^ In NP analysis, light scattering from dispersed particles may contribute to apparent absorbance in optical concentration detectors, particularly UV–vis, leading to an overestimation of concentration and distortion of the relative abundance of coexisting populations. Due to scattering effects, UV–vis detection tends to overestimate large particles, making their quantification infeasible and leading to artificially high mass percentages of the larger-particle/aggregate fraction.^29^ In contrast, the dRI response factor is governed by the refractive index increment (d*n*/d*c*) of the analyte, which tends to remain nearly constant for samples with comparable chemical composition, thereby enabling more reliable quantification of relative particle fractions.^30^

Multiangle light scattering (MALS) is an absolute detection technique that, when coupled to size-based separation methods, provides direct access to structural parameters of molecules and NPs without the need for calibration standards. Combined with an accurate determination of sample concentration and a known d*n*/d*c*, MALS enables the measurement of the MW, a value that reflects the average mass of the scattering entities and supports quantitative comparisons among different formulations or batches. Independently of concentration and without assumptions on particle shape or conformation, MALS also yields the root-mean-square radius or radius of gyration (*R*_g_), which describes the mass distribution around the particle center of mass. MALS enables determination of *R*_g_ across a size range of 10 to 500 nm, which can extend to 1 μm when appropriate shape-dependent models are applied and adequate angular resolution is available.^31^ Measurements approaching the lower end of this range, corresponding to small NPs, may be affected by reduced scattering intensity and decreased signal-to-noise ratio, which can impact the robustness of *R*_g_ determination.^32^ The joint analysis of MW and *R*_g_ distributions supplies additional structural information, allowing inferences on particle density and overall conformation.^33^ DLS, which can be applied both in-batch and in online configurations, provides complementary data measuring the Stokes radius or hydrodynamic radius (*R*_h_). When implemented in an online configuration, the accessible *R*_h_ range is generally more limited (∼0.5–300 nm) than in the in-batch mode. As in conventional DLS, the measured *R*_h_ depends on instrumental parameters such as the detection angle. In addition, in online configurations, the measurement can be affected by the flow rate and by the hydrodynamic conditions within the detection volume.^34^

MS-based detection in hyphenated platforms includes electrospray ionization (ESI)-MS strategies, which are predominantly established in combination with SEC and AF4 for the molecular and structural characterization of proteins, protein assemblies, and biopharmaceuticals. Their application to NP systems is generally indirect and typically relies on the analysis of separated fractions or specific nanomedicine-related components rather than on intact particles. For nanomaterials containing inorganic components, inductively coupled plasma MS (ICP–MS) provides elemental analysis and quantification. When coupled to separation techniques, ICP–MS enables the characterization of particle size distributions (PSD) while simultaneously measuring the elemental content. This hyphenated approach allows differentiation between dissolved ions and intact NPs, providing both structural and quantitative information.^35^

The online coupling of AF4 with NTA was also reported and enabled accurate, size-resolved particle counting of distinct NP populations within complex mixtures, a capability not achievable by AF4 or NTA when used alone.^36^

The real power of these detection techniques emerges in the multidetector (MD) approach, where various detectors are coupled in series (dRI and/or UV and/or FLD for concentration, MALS for *R*_g_, and DLS for *R*_h_). This configuration enables the simultaneous determination of multiple parameters, yielding a multidimensional picture of NP properties. The online coupling of MALS and DLS enables the determination of the Burchard-Stockmayer shape factor ρ (defined as the ratio *R*_g_/*R*_h_), which can provide useful qualitative information on the internal mass distribution and morphology of nanocarriers.^19,37^ This parameter has been reported as a useful descriptor for comparative structural assessment of different NP architectures, with literature examples suggesting different ρ ranges for systems such as hollow spheres, drug-loaded liposomes, and dense-core NPs including lipid NPs (LNPs).^37–39^ In this context, ρ may reflect differences in particle compactness and internal organization, thereby supporting the evaluation of cargo loading and structural integrity. However, the interpretation of absolute ρ values is strongly model-dependent and should be considered with caution, particularly for heterogeneous or deformable NP systems. Accordingly, while this parameter can provide valuable comparative structural insights, it does not allow unambiguous morphological classification of complex nanocarriers.^37–39^

The combination of dual concentration detectors, typically UV and dRI, permits the deconvolution of the chemical composition across the molar mass distribution of heterogeneous, two-component systems. This approach is particularly effective for characterizing species such as protein conjugates or copolymers, provided that the constituent components exhibit significantly different d*n*/d*c* and extinction coefficients (ε).^31,40^ In NPh formulations, this strategy can be extended to quantify drug loading by deconvoluting the respective contributions of the carrier matrix and the encapsulated species.^28,41^

Overall, the choice of detector or, more effectively, their integration in an MD system, critically influences the depth and reliability of the obtained information, upgrading AF4 and SEC from purely size-based separation tools into platforms for comprehensive NP physicochemical characterization. Figure 2 shows the range of information obtainable from each detector and from their combination.

**Figure 2:**
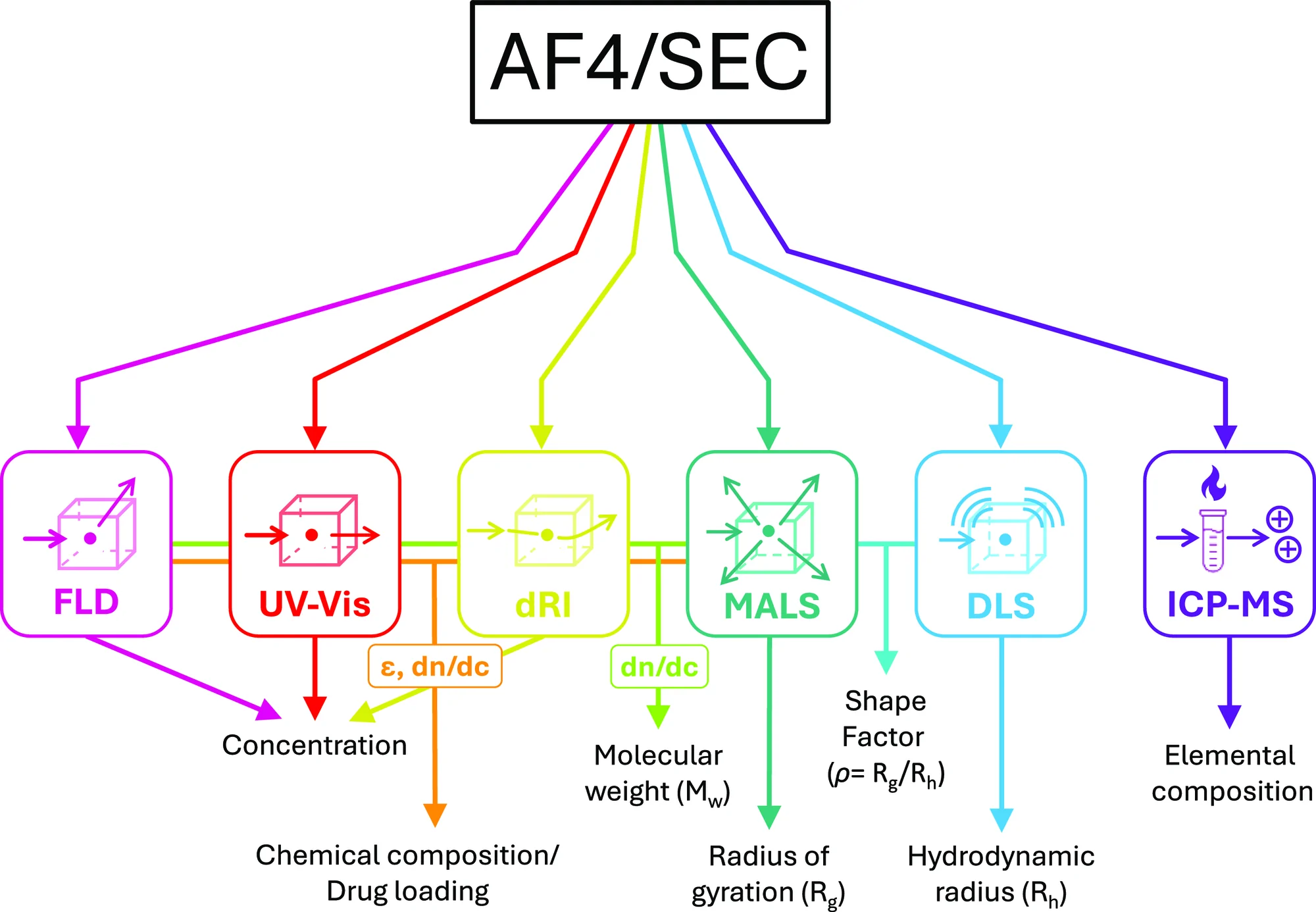
Principal detectors coupled to AF4 and SEC in NP analysis and the complementary information obtained from single and multidetector configurations.

## Comparative Evaluation of AF4 vs SEC

This section compares SEC and AF4 in terms of size-range resolution, capability to resolve subpopulations and aggregates, preservation of NP integrity, recovery, and interaction effects. Key operational aspects, including sample requirements, method development, throughput, and robustness, are also considered in the context of their practical applications.

### Size Range and Resolution

The different mechanisms of AF4 and SEC result in different ranges of applicability. The absence of a stationary phase makes AF4 suitable for the analysis of NPs over a wide separation range, from a few nanometers up to more than 1 μm.^19,42,43^ This range aligns well with the requirements for NPh characterization, establishing AF4 as one of the leading separation techniques in the field. In addition, AF4 provides high size resolution and can resolve NP populations with relatively small differences in hydrodynamic diameter, although the achievable resolution strongly depends on particle characteristics and operating conditions, including the crossflow profile and channel configuration.^44^ Over the past 10 years, advancements in channel design, such as optimized channel dimensions through miniaturization^45^ and automated crossflow gradients,^46^ have further improved the resolving power of AF4. As an example, in 2021, a commercially available short channel (14 cm length) was proposed to improve efficiency by reducing nonequilibrium band broadening. Under the specific experimental conditions investigated, this resulted in improved resolution of NP populations compared with a standard channel (27 cm length).^47^ A theoretical discussion on how channel dimensions and geometry affect separation performance in AF4 can be found in the literature.^48,49^

In contrast, the separation window in SEC is defined by the pore size of the stationary phase, which should generally be about three times larger than the hydrodynamic radius of the analyte to allow effective separation.^50^ Over the past decade, SEC columns with pore sizes around 300 Å have been widely used to characterize monoclonal antibodies and related biomolecules. Subsequently, larger-pore columns (∼500 Å) were introduced to allow the analysis of adeno-associated virus (AAV)-related products, which range around 20 nm. The analysis of larger particle size necessitates SEC columns with ultrawide pores,^51,52^ which, however, present additional challenges related to column particle stability, since the stability of a packed bed strongly depends on the average pore diameter.^53^ Particle size represents another critical parameter. The production of mechanically stable packing materials that combine very large pore sizes with small particle diameters remains technically challenging. From a geometrical perspective, the maximum analyte size that can be effectively fractionated is approximately one-third of the packing particle diameter; thus, columns packed with 2.5 μm particles are theoretically limited to analytes in the 250–300 nm range^50^ (the nature and deformability of the analyte must also be taken into account). Moreover, the use of smaller particles increases the shear stress experienced by biomolecules, potentially affecting structurally delicate systems.^50^ A theoretical study by Gritti investigated the transient diffusion of large biomolecules within fully porous sub-3 μm UHPLC particles and showed that very large analytes such as virus-like particles (VLPs, ∼50 nm) exhibit extremely slow intraparticle diffusion.^54^ Even when considering stationary phases with large pore size (∼900 Å), the model predicts that less than 40% of the column capacity would be effectively utilized, as VLPs cannot efficiently access the internal porous volume during the chromatographic time scale. These results highlight the intrinsic mass-transfer limitations of conventional porous chromatographic media for nanoscale bioparticles and suggest that improved separations would necessarily require stationary phases with significantly larger pores or alternative materials such as nonporous or superficially porous materials.^54^

Only recently have columns with 2.5–3 μm particle size and 1000–2000 Å nominal pore diameters become available, enabling the analysis of NPs with hydrodynamic diameters up to 100–200 nm. In 2023, Goyon et al. used prototype silica-based ultrawide pore size columns, with average pore sizes around 1300 Å, to analyze a series of LNP formulations ranging from 75 to 200 nm, showing good agreement between SEC retention times and DLS measurements.^53^ To date, various suppliers, including Waters (GTxResolve Premier BEH SEC 1000–2000 Å), Agilent (Bio SEC-5 1000 Å), Sepax (SRT SEC 1000/2000), Tosoh (TSKgel G6000PWxl/G7000PWxl), and Thermo Fisher Scientific (selected SurePac Bio columns), offer wide-pore SEC phases compatible with advanced detection techniques such as MALS and DLS, and have released application notes reporting their successful use for the analysis of NPs such as LNPs and VLPs.

In SEC, the range of analyte sizes that can be analyzed within a single separation can be enhanced by column configurations that either couple multiple columns packed with particles of different pore diameters in series or blend particles with varying pore sizes within a single column.^50^ An additional and particularly promising strategy is the development of columns featuring a gradient of pore sizes along the separation path; however, such designs remain technically challenging.^55^

### Separation of Subpopulations and Aggregates

As separative techniques, both AF4 and SEC offer the advantage of investigating particle size heterogeneity by enabling fractionation and differential detection of subpopulations. This is in contrast to ensemble methods such as in-batch DLS, which, while remaining the reference method for fast and easy-to-perform particle size determination, is strongly affected by the presence of multimodal or polydisperse samples. In fact, DLS tends to be biased toward larger objects, since a few strongly scattering species can dominate the signal and mask smaller populations, limiting the reliability of the derived PSD; a pitfall already underlined by regulatory bodies.^16,56–59^ Caputo et al. demonstrated that in-batch DLS analysis of clinical-grade liposomes loaded with a topoisomerase I inhibitor indicated a single, apparently monodisperse population (size of ∼110 nm, polydispersity index (PDI) < 0.05), whereas AF4-MALS revealed a more complex and polydisperse sample composed of two distinct populations that in-batch DLS failed to resolve.^37^

Unlike other techniques used in the field, both AF4 and SEC not only allow the separation of sample components but also enable the physical collection of fractions, which can subsequently be characterized by complementary techniques such as NTA^60^ or electron microscopy.^61^ MS-based approaches (e.g., ESI-MS/HRMS) are mainly used for the molecular characterization and quantification of components contained in or associated with the separated fractions (e.g., lipids, proteins, or other biomolecules) rather than for the direct detection of intact NPs.

When discussing heterogeneity, an important aspect to investigate in NPh characterization is aggregation, since even subtle changes in aggregation state can markedly influence NP physicochemical properties, stability, and overall performance in pharmaceutical applications.^62^ The wide size range accessible by AF4 enables the investigation of aggregate formation, as well as the detection of impurities and larger particles. SEC, on the other hand, provides high-resolution separation in the lower size range, making it especially suitable for characterizing early stage aggregation and for detecting small aggregates formed by NPs with a relatively small diameter. A representative case can be found in the complementarity of SEC and AF4 in the characterization of AAVs, virus-derived particles with highly uniform dimensions of 20–25 nm. In a specific comparative study reported in an application note,^29^ SEC has been shown to provide better resolution than AF4 for separating monomers from dimers, allowing their accurate quantification, especially when coupled to MALS detectors. However, large aggregates may be altered or removed during SEC separation, and for AAV samples containing such aggregates, AF4 represents a more appropriate alternative for separating and quantifying aggregates.^63,64^ The same limitation of SEC was observed in the analysis of different LNP formulations by Parot et al.^65^ In this study, SEC separation with a TSKgel-G6000PWxl column (Tosoh Biosciences, pore size >1000 Å) failed to characterize batches (representative separation is reported in Figure 3B) in which AF4 revealed differences in the presence of large aggregates (>200 nm, Figure 3A). Notably, such large aggregates were not eluted from the SEC column, suggesting that they were not merely excluded from the column pores, but were degraded during separation and/or retained by the column or frit packing acting as a physical filter.

**Figure 3:**
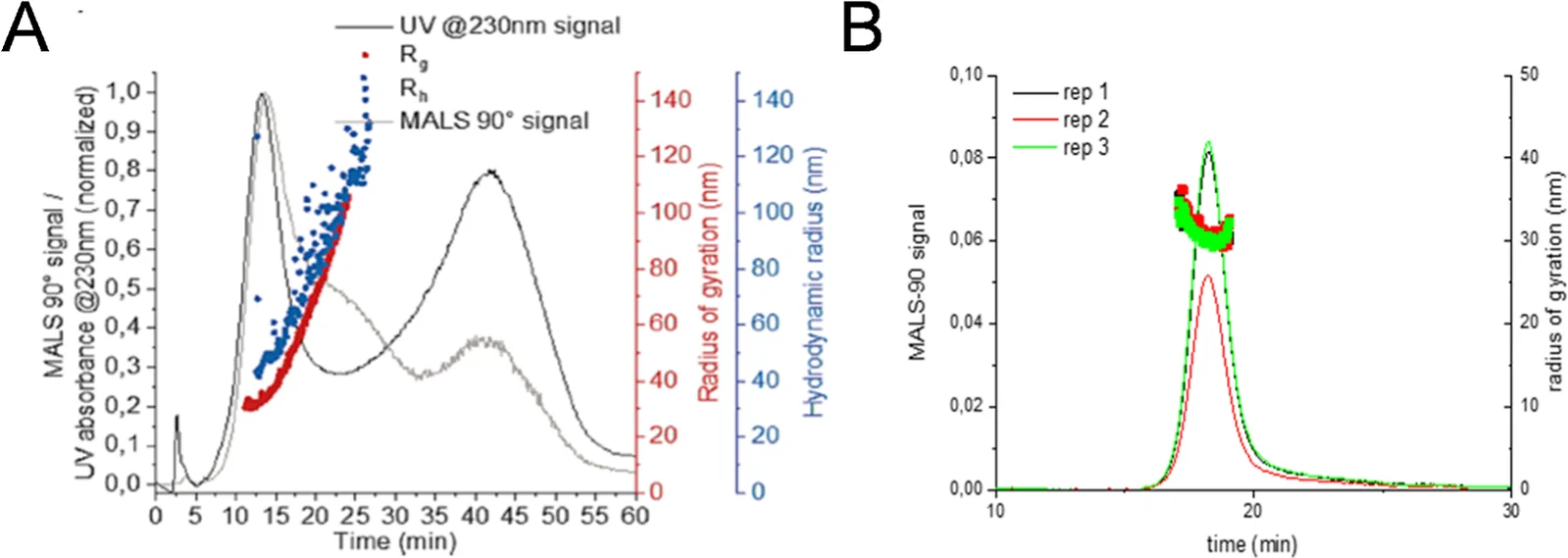
AF4 (A) and SEC (B) analysis performed on the same LNP batch. Adapted from ref 65. Available under a CC-BY 4.0 license. Copyright © 2024 The Authors. Published by Elsevier B.V.

This naturally leads us to the next sections since the ability of an analytical method to detect populations and aggregates inherently depends on its capacity to preserve supramolecular assemblies without causing irreversible retention or loss.

### Preservation of NP Integrity (Supramolecular Structures and Aggregates)

Separative techniques should be able to address the size heterogeneity of NPs without altering it. SEC, due to the presence of the stationary phase, may expose fragile NPs or weakly associated aggregates to shear forces and surface interactions that can compromise their native state. However, method parameters can be optimized to improve the suitability of SEC for the analysis of fragile NPs. For instance, to minimize interstitial shear stress while maintaining low backpressure, some authors suggest that columns with a wide internal diameter (7.8 mm) and particle sizes ≥5 μm should be preferred.^30^

In contrast, AF4 is generally considered to be gentler and more likely to preserve the integrity of NPs and their supramolecular assemblies. On the basis of this, SEC is best suited for relatively stable NPs or limited-size stable aggregates, whereas AF4 is more appropriate for fragile, multimodal systems and for preserving larger assemblies.

While this general picture can be drawn, it is important to note that AF4 is not exempt from stability issues, as fragile particles may be destabilized during separation due to dilution effects or the application of an excessively strong cross-flow. Nevertheless, recent instrumental configurations have been proposed to mitigate these limitations. Among them, Mildner et al. proposed a frit-inlet (FI) AF4 channel, enabling hydrodynamic relaxation of the sample upon channel entry and eliminating the need for a conventional focusing step, thereby reducing focusing-related sample losses and membrane interactions (Figure 4).^38^

**Figure 4:**
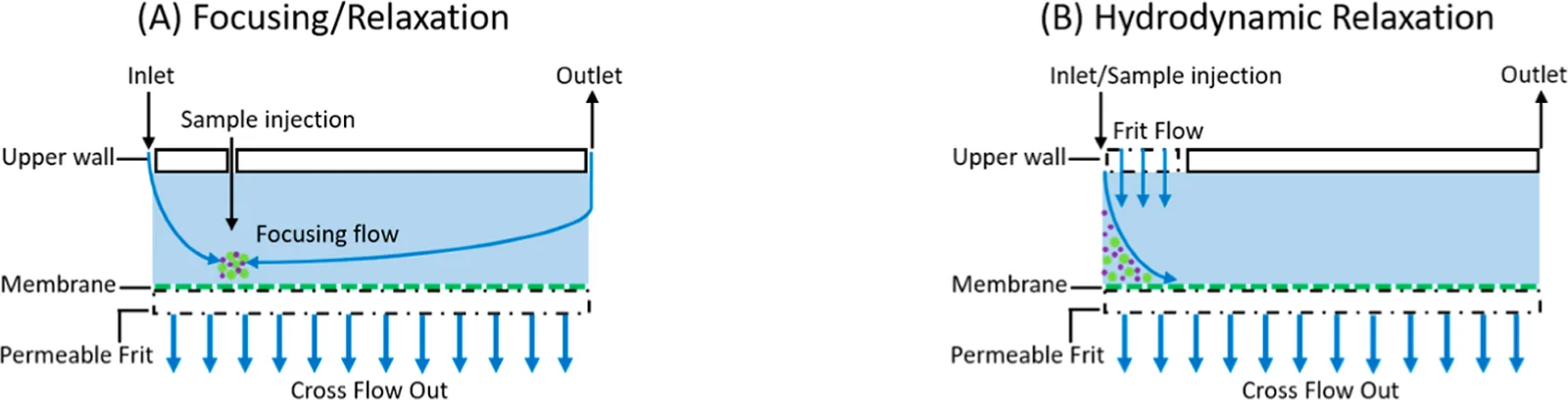
Representative diagram of relaxation and elution process in AF4: (A) focusing relaxation in conventional AF4; (B) hydrodynamic relaxation in frit-inlet or dispersion AF4. Reproduced from ref 31. Available under a CC-BY 4.0 license. Copyright © 2023 The Authors. Licensee MDPI, Basel, Switzerland.

The ability of AF4 to preserve NP integrity also depends on both the physicochemical nature of the NPh and the properties of the membrane material. Gao et al. compared regenerated cellulose (RC) and poly­(ether sulfone) (PES) membranes in the AF4 separation of mRNA-LNPs, finding that the PES membrane affected the structural integrity of the LNPs. This was confirmed by an increase in the peak area of the free mRNA signal, possibly due to the detrimental interactions between PES and the LNPs, causing drug release.^58^ Accordingly, careful selection of the membrane and optimization of the experimental conditions are required in AF4 to mitigate these critical effects.

### Sample Recovery and Interaction Issues

Another critical aspect in both AF4 and SEC is sample mass recovery (i.e., the ratio between the detected and the injected mass). Sample loss due to particle–membrane/stationary phase interactions is a major source of low recoveries and compromised quantitative reliability.^39^

In AF4, sample losses may occur during the focusing/relaxation step due to adsorption onto the membrane that serves as the accumulation wall and/or particle aggregation.^31^ In contrast, membrane-related losses are generally negligible once the elution step has started.^38^ This limitation can be mitigated by using FI-AF4 (see the previous section), which represents a suitable alternative for samples that are susceptible to undesirable membrane interactions.

Additionally, recovery in AF4 strongly depends on the membrane material and on the sample characteristics, with positively charged particles being more severely affected. Indeed, the most common membrane material is RC, which has a p*K*_a_ of ∼3.5 and is therefore negatively charged at neutral pH, promoting electrostatic interactions.^66^ Although systematic investigations exist for protein-based systems,^67^ similar comprehensive studies focused on NP recovery are still relatively scarce. Consequently, recovery assessments should be performed on a case-by-case basis for specific formulations.^16,58,68^ In the analysis of mRNA-LNPs, recovery was found to depend strongly on the lipid composition, even under constant method conditions.^58^ A detailed investigation of the factors that should be carefully considered for reliable calculation of percentage recovery, including those affecting both sample recovery and total recovery, has been reported.^68^ The study highlights the dependence of recovery on the nature and complexity of the sample and its impact on PSD determination. Finally, eluent composition, including ionic strength, salt type, and the presence of surfactants, can also affect retention behavior.^69^

In SEC, sample recovery is influenced by adsorption and/or potential retention of NPs by columns or frit packing, which can act as a physical filter. Consistently, SEC analyses of NPh samples containing a significant fraction of aggregates might be associated with lower mass recovery.^65^ Recovery is strongly dependent on the nature of the NPs and the column used. As early as 2012, Zhang et al. systematically investigated the influence of stationary phase chemistry and pore size on the SEC separation of different siRNA–LNP formulations. Among the columns tested, the cationic-modified TSKgel G6000PWxl-CP (Tosoh Biosciences) provided superior resolution and minimized secondary interactions, likely due to electrostatic repulsion between the positively charged resin and the LNP surface.^70^ In liposome analysis, recovery was found to be column-dependent when comparing G5000PWxl (Tosoh Biosciences, 10 μm) and G6000PW (Tosoh Biosciences, 17 μm), despite similar stationary phases, with a more pronounced effect for drug-loaded formulations. The lower recovery observed with the G5000PWxl column, particularly for drug-loaded systems, was attributed to increased membrane rigidity following drug incorporation into the lipid bilayer. The enhanced rigidity likely hinders passage through the smaller interstitial spaces of the more tightly packed particles.^30^

In recent years, significant progress has been made in column hardware and surface engineering strategies aimed at minimizing nonspecific interactions. For example, the use of hydrophilically modified hybrid organic–inorganic column hardware (based on BEH-like siloxane chemistry–hydrophilic hybrid surface technology, h-HST) has been demonstrated to significantly reduce unwanted nonspecific interactions.^71^

### Sample Requirements, Sensitivity, and Overloading

Sample requirements and sensitivity are critical aspects when applying size-based separations to NPhs, particularly in pharmaceutical settings, where sample availability can be limited. SEC performs well with relatively small sample amounts but is highly sensitive to overloading, which can cause peak distortion, altered retention, and reduced resolution, particularly for polydisperse or aggregated samples. These constraints can limit sensitivity when probing low-abundance populations or subtle size distribution features and have motivated regulatory guidance recommending orthogonal methods to support SEC data interpretation.^72^

AF4 generally tolerates higher injected masses, although overloading during focusing can still affect separation through enhanced particle–particle or particle–membrane interactions. In AF4, overloading can be mitigated by reducing crossflow, optimizing flow conditions to focus the sample in wider channel regions (i.e., adjusting the flow rate ratio to position the focusing zone near the wider channel inlet, thereby reducing local mass density), or using larger channels.^16,73,74^ While AF4 can accommodate different injected masses, its absolute sample requirements relative to SEC depend strongly on a combination of detector sensitivity, channel geometry, injection/focusing conditions, and sample recovery.^19^ Notably, sample consumption can be significantly reduced by implementing alternative or miniaturized formats; for instance, hollow-fiber flow FFF (HF5) offers drastically lowered sample volume requirements and improved mass detection limits due to its miniaturized channel volume.^75^

In both techniques, the achievable sensitivity ultimately depends on the detector used, but overall, SEC favors applications where sample amounts are limited, provided that overloading effects are carefully controlled, while AF4 offers greater flexibility in injected mass with reduced common overloading consequences.

### Method Development, Throughput, and Robustness

From a methodological perspective, SEC is often regarded as a relatively straightforward technique. Method optimization in SEC is largely dictated by column technology, particularly pore size distribution and stationary phase chemistry, while the mobile phase composition represents the primary experimental variable to be adjusted. In most applications, mobile phase optimization aims to preserve NP stability and minimize nonspecific interactions; guidance on its selection can be found in the literature^20,76^ or in column user manuals. Consequently, SEC method development is typically rapid and relies on a limited number of critical parameters.

In contrast, method development in AF4 requires broader and more flexible optimization of experimental conditions based on the NP subclass and, in some cases, specific formulation or intended application. Additionally, AF4 is governed by multiple interdependent parameters, including crossflow magnitude and profile, focusing or relaxation conditions, membrane material, carrier composition, channel geometry, and instrument hardware.^39^ The extensive method optimization of these parameters often requires a deep understanding of the separation mechanism and structured optimization strategies, including multivariate or design-of-experiments approaches, and is supported by the availability of methodological guidelines.^16^

In terms of analytical throughput, SEC generally enables shorter analysis times (within 15–30 min) and requires minimal system equilibration between runs, making the technique well suited for high-throughput applications such as quality control.

AF4 is generally associated with longer analysis times, often exceeding 30 min, particularly when conditions are carefully optimized to preserve NP integrity, such as using gentle cross-flow. However, strategies such as gradually decreasing the crossflow during elution can release particles of incrementally larger sizes, thereby speeding up the separation process.^38^ Additional factors, including channel equilibration and membrane conditioning, further contribute to the overall analysis time. Consequently, AF4 is more commonly employed for in-depth characterization studies, where resolution and structural information are prioritized over the sample throughput.

Robustness represents another key aspect, particularly when considering the method reproducibility and transferability. SEC methods are generally robust and highly reproducible in conventional applications,^20^ with performance largely dictated by column properties and well-controlled chromatographic parameters. Once established, SEC methods tend to exhibit stable retention behavior and are relatively insensitive to minor variations in operating conditions, facilitating the method transfer across instruments and laboratories. While robustness has been demonstrated for certain NP classes, such as AAVs,^71,77^ systematic evaluation of SEC method robustness and reproducibility for less studied NPh formulations is still required.

AF4 methods, while highly robust when properly optimized, are more sensitive to variations in experimental conditions because of the number of parameters involved in the separation process. Factors such as membrane batch-to-batch variability, channel assembly, system cleanliness, and gradual membrane fouling can significantly influence the separation performance, recovery, and repeatability.

Therefore, successful method transfer relies on well-defined and harmonized operating procedures as well as on appropriate system conditioning and quality control criteria. Recent collaborative efforts, including those performed within the European Nanomedicine Characterization Laboratory (EUNCL) and the National Cancer Institute Nanotechnology Characterization Laboratory (NCI-NCL), have shown that AF4 measurements of complex NPhs, such as liposomal formulations, can achieve optimal interlaboratory reproducibility, supporting the potential of AF4 for reliable quality control applications.^37^ Furthermore, the availability of standardized approaches, including the ASTM E3409–24 standard test method for the analysis of liposomal drug formulations using MD AF4,^78^ recognized by the Center for Drug Evaluation and Research (CDER) of the U.S. FDA in 2025, further supports the growing maturity and standardization of AF4 in nanomedicine characterization.

The practical robustness and transferability of AF4 methods are also influenced by the current instrumentation landscape, which is dominated by two commercial platforms from Wyatt Technologies and Postnova Analytics. While this has facilitated methodological consolidation and detector integration, it may also limit broader cross-platform standardization compared to SEC, which can be performed on any chromatographic system.

A comparison of the strengths and limitations of AF4 and SEC for NP size characterization is presented in Table 1.

**Table 1:** Comparative Analysis of the Strengths and Limitations of AF4 and SEC for NPh Size Characterization

parameter	AF4	SEC
separation principle	separation based on particle diffusion in an asymmetric flow field perpendicular to the channel	separation based on size exclusion through a porous stationary phase
size range	broad size range, typically from a few nm up to ∼1 μm	limited by column pore sizes (typically <200–300 nm)
size resolution	high; capable of resolving heterogeneous and multimodal populations	good for relatively small systems, but limited by column porosity
suitable samples	applicable to a wide range of NPs, including soft and fragile systems if proper setup is applied (e.g., FI-AF4)	mostly suitable for rigid and structurally stable NPs; applicability to soft nanomaterials is still under investigation
particle integrity	generally preserved due to the absence of a stationary phase, although stability during focusing and separation should always be verified	potential mechanical stress, shear, or interactions with the stationary phase may affect particle integrity, highly depending on the nature of the analyte
sample recovery	highly method- and sample-dependent; generally good; membrane material selection is critical and frit-inlet configurations may be beneficial	highly dependent on NP properties and column properties
sensitivity/sample amount	highly format- and method-dependent; standard channels typically require higher absolute mass, but miniaturized or alternative formats (e.g., HF5) drastically reduce sample consumption	generally requires lower sample amounts, but is susceptible to overloading, which depends on column dimensions, injection volume, and sample concentration
analysis time	variable; typically, longer than SEC (30–60 min)	usually shorter (15–30 min)
method development and optimization	often time-consuming due to multiple interdependent parameters and sample-dependent optimization	generally faster due to fewer adjustable parameters
cost and operational complexity	higher instrumentation cost and specialized expertise required	lower cost and easier routine operation
robustness and method transferability	more sensitive to experimental parameters; careful control and harmonization of operating conditions are required to ensure interlaboratory reproducibility and method transferability	generally robust and transferable for established applications, although transferability for complex NP systems still requires systematic evaluation

## Application of AF4 and SEC to NPh Characterization

Each class of NPhs has distinct structural features that influence not only their pharmaceutical applications but also their analytical characterization and associated challenges. Therefore, the general considerations outlined above should be interpreted in the context of the specific NPh class under analysis where analytical methodologies enable the identification and characterization of class-specific features.

In this section, applications of AF4 and SEC will be compared with respect to the representative NPh classes. The focus is on NP-based drug delivery systems for which AF4 and SEC provide established or emerging analytical value, prioritizing representative classes that best illustrate the performance and limitations of these size-based techniques rather than attempting a comprehensive coverage of all NPs systems. To this end, NPhs will be classified as lipid-based, virus-based, polymeric, and INPs. The section provides a description and discussion of the applications reported during the period 2020–2026, with the aim of providing the reader with a critical overview of current research trends in this field.

### Lipid-Based NPs

Lipid-based NPs represent a broad and heterogeneous class of nanocarriers widely employed in drug delivery, which encompasses several distinct classes, including liposomes, LNPs, solid lipid–based systems such as solid LNPs (SLNs) and nanostructured lipid carriers (NLCs), and lipid nanocapsules (LNCs).^79^ Another lipid-based class of NPs is represented by extracellular vesicles (EVs), which are emerging as promising tools for drug delivery.^80^

The various types of lipid-based NPs differ in internal organization, lipid composition, and physicochemical properties. However, despite these structural differences, they share common analytical challenges, including soft and dynamic architecture, formulation-dependent size distributions, and susceptibility to aggregation or structural rearrangements under nonphysiological conditions. In this section, AF4 and SEC applications to liposomes, LNPs, and EVs will be discussed. Other nanocarrier platforms relevant to drug delivery, such as SLNs, NLCs and LNCs, have been less explored using size-based separation techniques. In these systems, AF4 has been reported in isolated cases, often coupled to MD setups for detailed PSD analysis,^81^ whereas SEC is predominantly employed for carrier–drug separation or purification rather than dimensional characterization.^82–84^

### Liposomes

From a pharmaceutical perspective, liposomal formulations represent the most mature and clinically validated systems, with Doxil (liposomal doxorubicin) being the first FDA-approved NPh in 1995.^79^ Several liposome-based products are currently approved for clinical use, primarily to improve pharmacokinetics and reduce systemic toxicity of small-molecule drugs.

Liposomes are composed of one or more lipid bilayers surrounding an aqueous core and are characterized by a well-defined vesicular architecture and relatively low sensitivity to dilution- or shear-induced rearrangements. Most liposome-based pharmaceutical formulations fall within the 50–150 nm size range.

Both SEC and AF4 have been employed for the characterization of liposomal drug carriers. SEC has traditionally been used for purification purposes^20^ and determination of drug EE.^85^ Multiple reports note that the main issue in SEC of liposomes, using earlier-generation materials, is lipid/liposome adsorption to the stationary phase, leading to low recovery. Presaturation of columns with lipids or empty liposomes was proposed as an approach to mitigate these effects, with the risk of introducing contamination.^85,86^ The introduction of new-generation columns significantly improved SEC performance in liposome analysis. Recently, by employing a TSKgel G6000PW column (Tosoh Biosciences; pore size >1000 Å), Tang et al. developed a semiquantitative SEC-UV-MALS-capillary inline viscometer (IV)-dRI method for the characterization of shape heterogeneity in liposome–lipodisk mixtures (with *R*_g_ ranging from 20 to 50 nm). The column enabled resolution of liposomes (spherical) and lipodisks (nonspherical) when their diameters differed by approximately 3-fold. Despite their different shapes, when the two populations exhibited comparable sizes, SEC alone was insufficient to resolve morphologically heterogeneous mixtures. In such cases, MALS measurements provided the additional dimensional information required to distinguish particles of different shapes based on the scaling relationship between *R*_g_ and MW.^30^ Interestingly, the combination of the increased size selectivity of new-generation columns with the exponential correlation between *R*_g_ and MW obtained by light scattering offers a valuable tool for properly defining shape heterogeneity during early stage formulation development and quality control. This methodology may be broadly applicable to other types of lipid-based or polymer-based nanostructures as the correlation was found to depend only on size and morphology.

Size distribution of liposomes is established mainly by AF4.^78,87–90^ In 2020, Parot and co-workers developed a validated best-practice protocol based on MD-AF4 for the comprehensive physical characterization of liposomal formulations.^39^ Using liposomal doxorubicin hydrochloride as a prototypical test material, they optimized key operational parameters, including fractionation quality, recovery, and separation efficiency, to ensure accurate measurements of particle size, distribution, and shape. The authors point out critical points encountered and discuss best practice notes for their management, giving a useful theoretical and practical guide for users. For proper recovery calculations, the unretained peak should be included, and it is recommended to consider its absorbance/signal (i.e., fluorescence) properties that differ from those in the formulation; to improve recovery, passivation of the membrane with a suitable coating (surfactant, protein) is recommended. Experimental parameters such as mobile phase composition and injection volumes have been brought to attention: polyethylene glycol (PEG)-modified liposomes are not sensitive to PBS source and pH fluctuations; given its importance for the fractionation process and detector response, injected mass must be controlled, while volume can be varied as the focusing process was shown to be effective for low-concentration samples. For proper polydispersity characterization, as the angle of DLS measurement affects the measured *R*_h_ in a size-dependent manner, calibration of the platform with size standards over a range suitable for the analyte is required, and to counterbalance the loss of detector sensitivity in dispersed mass samples, injected masses can be increased by taking advantage of the focusing process during injection of large sample volumes.^39^ Method robustness was confirmed through cross-validation across multiple laboratories, instrument platforms, and formulations, proving its suitability for preclinical R&D, industrial quality control, and regulatory assessment. The authors demonstrated that the validated method can be horizontally used with appropriate adaptations to a wide range of liposomes.^39^

One key feature for liposomes is the drug/lipid content determination related to the size distribution. In this context, AF4 fractionation and LC–MS analysis of collected fractions have been proposed as a promising approach to compare size distributions and drug/lipid content to support liposome development and comparability testing.^60^

Another important aspect is the assessment of stability and structural rearrangement under biorelevant or stress conditions. The use of SEC for this purpose may be limited by potential interactions between liposomes and the stationary phase, which can alter their structural integrity and potentially introduce separation-induced artifacts, while AF4-MALS-dRI proves to be suitable.^91^

### Lipid NPs

LNPs have emerged for their tremendous potential for advancing the delivery of therapeutic agents, particularly mRNA therapeutics, as has been clearly demonstrated by their central role in the rapid development and clinical success of COVID-19 vaccines.^58^

Despite structural similarities with liposomes, the distinct core structure of mRNA-LNPs, characterized by ionizable lipids electrostatically complexed with RNA and a relatively labile lipid monolayer shell, results in a more dynamic internal organization.^79^ Consequently, the direct translation of analytical protocols from liposomes to LNPs is often inadequate, necessitating specific methodological adaptations to preserve particle integrity. The inherent fragility and susceptibility of LNPs to destabilization can readily promote particle aggregation and reduced sample recovery, thereby introducing additional analytical challenges during size-based separation.^38^ A recent review discussing the challenges and advances in analytical separation techniques for RNA–LNP therapeutics, including carrier characterization, is available here.^92^

In this context, the open-channel configuration of AF4, preserving the integrity of the delicate LNP-RNA complex, allows the characterization of their size distribution and aggregation. However, substantial method optimization, such as the implementation of a frit-inlet channel, was required to minimize sample loss and particle aggregation, ensuring satisfactory reproducibility and acceptable sample recovery.^38^ Beyond size distribution, the integration of AF4 with an MD system provides a unique window into the LNP internal architecture. By determining the shape factor (ρ = *R*_g_/*R*_h_), AF4-MD can distinguish between the desired dense-core morphology and potentially inactive, hollow, or expanded structures. Consequently, AF4 serves not only as a sizing tool but as a sophisticated probe for the structural quality and batch-to-batch consistency of LNP-based therapeutics.^58,65,93,94^ To further expand AF4 analytical capabilities for LNP quantitative assessment, its coupling with NTA^65^ has been proposed. Additionally, the recent literature highlights an emerging trend toward coupling mainly AF4, but also SEC, with small-angle X-ray scattering (SAXS),^95–98^ a technique that can probe the internal structure of LNPs since the scattering length density of the nucleic acid component is significantly higher than that of lipids when exposed to X-rays. In a recent paper by Kolb et al., SAXS was initially used to establish a structural reference for interpreting MALS data. Once validated, the method can rely on MALS alone, making it applicable using standard AF4–MALS platforms within routine lab environments as part of the standard assays for better control of LNP quality.^97^

LNP sizing using SEC is, instead, possible but more challenging since the shear forces experienced within the stationary phase can compromise the structural integrity of these soft nanocarriers, potentially causing the disruption or dissociation of fragile aggregates. Nevertheless, Jia et al. showed the ability of a TSKgel G6000PWXL-CP column (Tosoh Biosciences) to provide size information on different LNP formulations ranging from 25 to 50 nm in radius. The SEC-MALS profiles demonstrated that, for the majority of each chromatogram, the radius decreased with retention time, suggesting that effective size-based retention was achieved.^99^ The method was applied to the determination of the distribution of RNA content as a function of the LNP-RNA size. By removal of the scattering contribution to the UV signal from the LNP particles and use of a correction algorithm implemented in the nanoconjugate analysis module of ASTRA software (Wyatt Technology), the UV signal at 260 nm was employed for payload quantification.

In 2023, Goyon et al. used a prototype silica-based ultrawide-pore size SEC column from Waters Corporation, treated via a two-step PEGylation silane reaction, with an average pore size of 1275 Å, to characterize a series of LNP formulations. The SEC elution times were plotted against the sizes determined by DLS (from 75 to 250 nm, Figure 5), showing an excellent correlation for most samples. Interestingly, deviations from this correlation were associated with differences in PEG-lipids used during LNP synthesis, suggesting that these discrepancies might reflect the tendency of DLS to overestimate NP sizes in the presence of heterogeneous and aggregated populations (a tendency influenced by the degree of PEGylation) rather than reflecting a limitation of SEC.^53^

**Figure 5:**
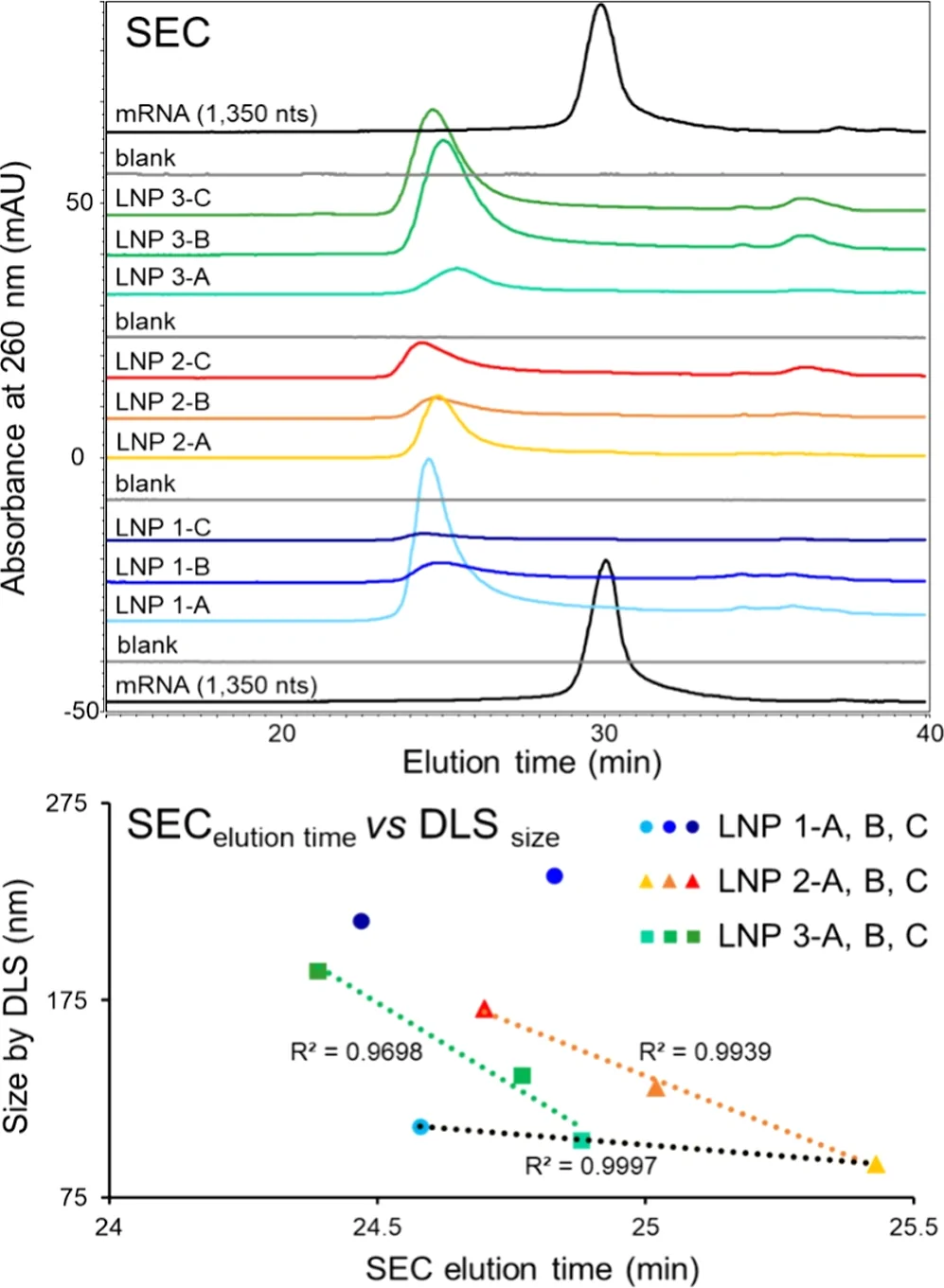
SEC-UV profiles of mRNA-LNP samples and the corresponding mRNA standard. The LNP sizes determined by DLS were plotted against the SEC elution time of the main peak. LNPs 1, 2, and 3 correspond to LNPs prepared with various PEG-lipid compositions. Each LNP was either unconjugated (“-A”) or conjugated to Fab 1 (“-B”) or Fab 2 (“-C”). Adapted from from ref 53. Available under a CC-BY 4.0 license. Copyright © 2023 The Authors. Published by American Chemical Society.

When facing heterogeneous mRNA-LNP formulations, Parot and co-workers showed that SEC separation on a TSKgel-G6000PWxl column (Tosoh Biosciences, >1000 Å) led to a significant underestimation of the PDI and a failure to detect large-diameter populations that are crucial for predicting in vivo toxicity and transfection efficiency (Figure 3).^65^ However, ongoing technological advancements are also improving this aspect of SEC. In the same paper mentioned above, Goyon et al. showed that the SEC method was able to resolve a prepeak preceding the main LNP peak, which, based on online MALS-dRI data, was attributed to the presence of particle aggregates.^53^

Another key quality attribute in mRNA-LNP characterization is the determination of the EE and the quantification of free mRNA in the final product, which are essential to assess the effectiveness of the loading process and/or the stability of the formulation. Gao et al. proposed an optimized AF4-MD platform able to resolve naked mRNA from mRNA-loaded LNPs through two sequential exponential crossflow decreases, facilitating the direct quantification of EE.^58^ Goyon et al. showed that SEC can also separate free mRNA and intact LNPs at different retention times, theoretically enabling the use of SEC to study encapsulation efficiency, although the authors did not fully explore this application.^53^

Finally, both techniques have also demonstrated suitability for studying the stability of loaded LNPs under near-physiological conditions, including in the presence of human plasma.^100,101^

Altogether, the literature demonstrates that the structural and compositional complexity of LNPs clearly requires thorough characterization, ideally relying on MD and multiattribute analytical platforms. In this framework, both SEC and AF4 can provide complementary insights, which must be critically interpreted and correlated with biological data to meaningfully understand their impact on functional performance and therapeutic efficacy.^59^

### Extracellular Vesicles

In addition to synthetic lipid-based nanocarriers, cell-derived nanosystems, such as EVs, represent an emerging class of biologically derived nanocarriers. EVs comprise a heterogeneous population of small lipid-based NPs (exosomes typically ranging from 50 to 150 nm) that play a central role in numerous physiological and pathological processes. Owing to their intrinsic targeting capabilities, they are increasingly investigated as natural nanocarriers for the delivery of therapeutic cargo to specific cells and tissues, as demonstrated by the large number of clinical trials with native or genetically modified EVs, postmodified EVs, or EV-inspired liposomes.^80,102^ From a drug delivery standpoint, EVs share structural similarities with liposomes; however, the compositional heterogeneity of their membrane lipids and associated proteins enhances tissue specificity while reducing nonspecific interactions and potential immunogenicity.^80,103^

Despite their advantages over synthetic nanocarriers, EVs face significant challenges for clinical translation. Key limitations include their intrinsic complexity, size heterogeneity, and batch-to-batch variability during production. Addressing EV heterogeneity is critical, with separation techniques such as SEC and AF4 playing a central role in ensuring sample consistency and integrity. One primary need for EV characterization is their isolation from cell supernatants or biological fluids. EV isolation is mainly performed by SEC, with the notable advantage of preserving EV functionality.^104^ In contrast, AF4 has rarely been employed as an EV isolation method. While a rigorous study published in 2019 first demonstrated the power of AF4 in the separation of exosome subpopulations in terms of particle morphology, size, and relative abundance through proper modulation of crossflow, channel height, and sample focusing,^105^ few applications have followed in recent years.^106^ A programmed field decay in AF4 successfully separated particles in the 20–200 nm range, though resolution decreased for sizes above 200 nm. A higher initial crossflow rate was required to achieve sufficient separation force and to distinguish small exosomes (∼30 nm) from contaminating proteins and small molecules.

AF4 has also been successfully coupled off-line to CE to add a second separative dimension.^107^ The 2D system can process large injection volumes and effectively separate EVs from smaller abundant impurities. This is particularly advantageous for the analysis of large-volume samples or samples with complex matrices. By first separating EVs from components of different sizes using AF4, the EV fractions enter the second separation stage (CE) with minimal contaminants, reducing the risk of CE overloading and improving the separation performance. The use of large-volume sample stacking (LVSS) and CE in a similar setup enhanced sample loading and improved EV recovery and resolution from high-abundance matrix components after AF4.^108^ Although interestingly implemented in a 2D analytical setup, AF4 serves only as an isolation step discriminating EVs ranging from 30 to a few hundred nanometers.

A recent study reports a critical comparison of different analytical methodologies to evaluate EV properties in terms of size, surface charge, and protein content and represents a systematic example of the application of cryo-TEM, NTA, AF4, and SEC for analytical separation and online MALS characterization of isolated EVs obtained by differential ultracentrifugation (dUC) or membrane affinity.^109^ The study highlights the limited performance of SEC-MALS in detecting large EVs, which were instead detected by AF4-MALS (up to 475 nm in dUC-EVs and 539 nm in membrane affinity-EVs; Figure 6A–D, respectively). It can be argued that the limiting factor is the SEC column pore size (1000 Å). Both AF4 and SEC are capable of separating EVs from other contaminants, but they remain complementary. Indeed, as depicted in Figure 6C,D, a difference was observed in the signals obtained by AF4-MALS and SEC-MALS for membrane affinity EVs. A potential explanation for the observed difference in signal intensity and noise is that the particles may have been disrupted during AF4, potentially due to the focusing step and the higher flow rates applied in AF4 compared with SEC (pump flow in SEC = 0.5 mL/min and in AF4 = 1.0–1.6 mL/min). Overall, both techniques allowed the discrimination of EV subpopulations and protein contaminants, with AF4 showing superior resolution capacity and the ability to detect larger particles and agglomerates.

**Figure 6:**
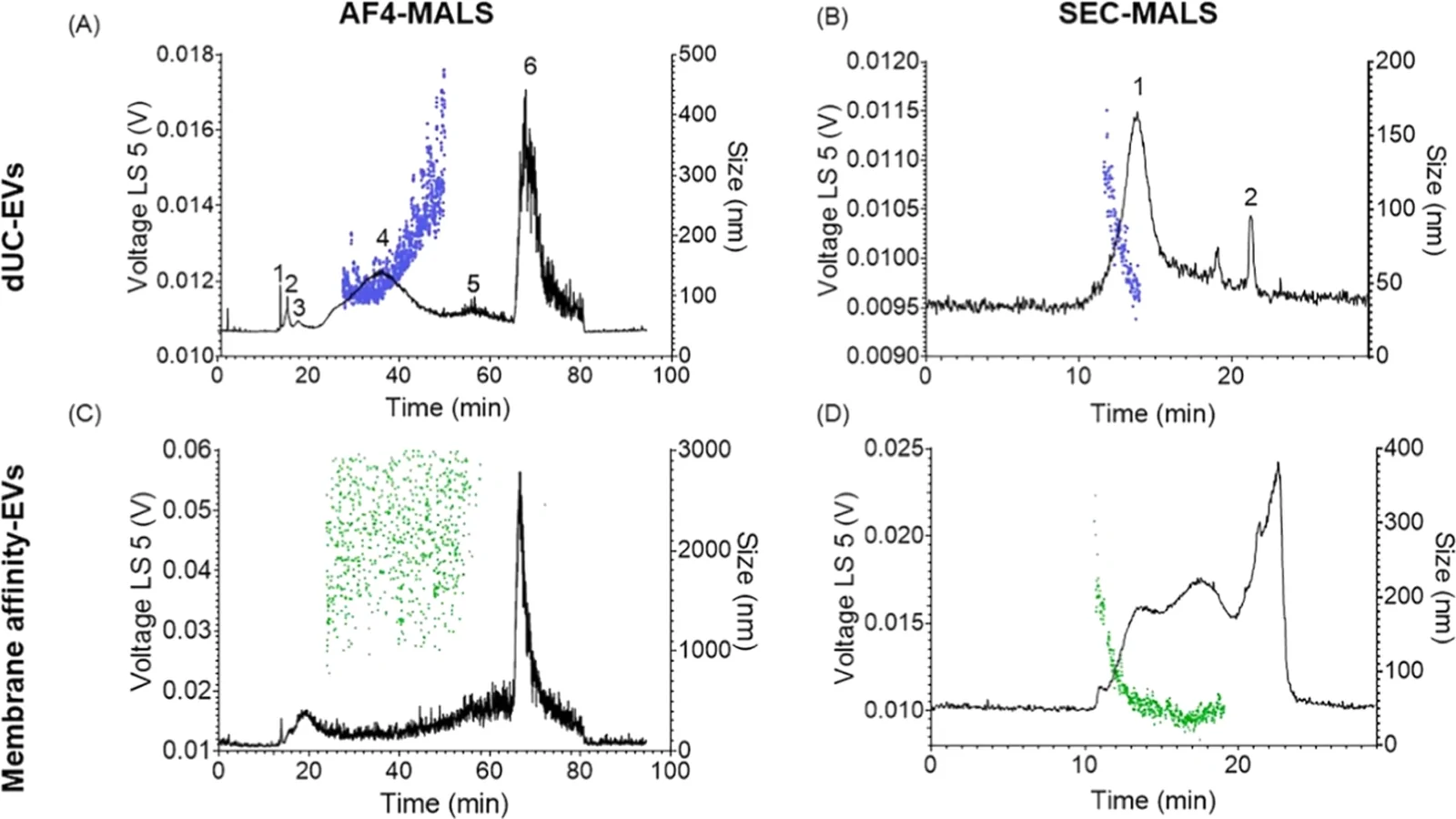
AF4-MALS profiles obtained for dUC EVs (A,B) and for membrane affinity EVs (C,D). Black lines represent the light-scattering signal at the detector at 90°; blue and green dots correspond to the size of EVs (rms × 2). Panel A: P#1: void peak; P#2 and P#3: protein contaminants and/or small particles; P#4: EVs <170 nm; P#5: larger EVs; P#6: bigger particles and agglomerates eluted when crossflow reaches 0 mL/min. Panel B: P#1: EVs; P#2: protein contaminants. Reproduced with permission from ref 109. Copyright © 2024 Elsevier B.V.

### Virus-Based NPs

With the increasing impact of gene therapy and vaccine development, virus-based particles, including recombinant AAVs (rAAVs) and VLPs, have become a versatile class of nanostructures with broad applications in the pharmaceutical field.^110^ Although AAVs and VLPs are classified from a regulatory standpoint as gene therapy products and vaccine biologics, respectively, rather than NPhs, they are included in this review due to their nanoscale dimensions and structural complexity, which make them highly relevant model systems for evaluating size-based analytical techniques.

### Recombinant AAVs

AAV vectors consist of a near-spherical protein shell containing a single-stranded DNA genome, with highly uniform dimensions of approximately 20–25 nm in diameter.^111^ The AAV capsid displays icosahedral symmetry and is constructed from 60 subunits derived from three viral proteins, VP1, VP2, and VP3, typically present in an approximate 1:1:10 stoichiometry.^112^ rAAVs used in gene therapy differ from wild-type AAVs due to the absence in their genome of the regions encoding replication and capsid formation, which eliminates their ability to replicate autonomously. rAAVs, which are sometimes classified as VLPs, have emerged as a leading platform for in vivo gene therapy due to their nonpathogenic nature, low immunogenicity, and broad tropism. The growing number of approved products and the more than 350 clinical trials underway worldwide,^112^ together with increasingly stringent regulatory requirements for comprehensive characterization, collectively underscore the need for robust and reliable analytical strategies to ensure the quality, safety, and efficacy of rAAV products.

Key CQAs of AAVs comprise physical titer (total AAV particle concentration), particle size, full-to-total capsid ratio, viral protein composition, post-translational modifications (PTMs), and stability (including aggregation and degradation tendency).^110,112^ Overviews of the analytical techniques commonly used for their characterization can be found in the literature.^63,110^ Among these, SEC plays a prominent role.

The main potential and application of SEC lie in its ability to study aggregation levels. Aggregation is a known degradation event for rAAV and is influenced by multiple factors, including serotype, capsid titer, empty-to-full particle ratio, formulation buffer, storage conditions, and thermal stress.^110^ Similar to protein therapeutics, aggregates in AAV products can lead to deleterious effects on transduction efficiency and immunogenicity.^112^ SEC for AAV analysis is currently carried out with columns and mobile phase conditions traditionally used for SEC of proteins ,and it has been extensively employed to achieve efficient separation of monomers, dimers, and higher-order aggregates in AAV samples, using either a UV detector or, for enhanced sensitivity, an FLD that exploits the intrinsic fluorescence of the particles.^113^ Recently, advanced column technologies that minimize nonspecific interactions (hydrophilic, diol-modified surface) and improve separation efficiency, thanks to 2.5–3 μm particles and 700 Å pores, have been shown to achieve selective and reproducible size characterization of AAVs in reduced analysis times.^71,114^

SEC, under optimized conditions, can offer high resolution in the separation of monomers from dimers, allowing accurate quantification.^29^ However, large aggregates may be altered or removed during SEC separation. Buff et al. evaluated six wide-pore SEC columns in the analysis of rAAV and found optimal selectivity using larger pore size (550–700 Å) columns. When applied to monitor an rAAV sample during a stress study over 3 days at 40 °C, consistent trends in the relative abundance (area %) of higher-order aggregates, dimers, and fragments were observed across all columns. Interestingly, the levels of higher-order aggregates increased significantly during the first 2 days before decreasing after 3 days. The authors attributed this unexpected behavior to the formation of insoluble particulates that cannot be detected by SEC, as they may be retained or adhere to the column frits.^115^

Recently, the combination of SEC with an additional chromatographic dimension and MS detection has been employed to further enhance its capability for multiattribute analysis. Aebischer et al. reported a SEC–UV–RPLC–MS workflow for the simultaneous assessment of aggregates, capsid identity, and PTMs, assessing structural integrity and stability of AAVs. In this approach, SEC is used to separate intact viral particles and quantify high-MW species, followed by heart-cutting of the monomeric fraction for downstream RPLC–MS analysis under denaturing conditions. The intact mass of viral capsid proteins can provide information on serotype identity and PTMs (such as acetylation and phosphorylation), revealing key insights into the tropism, infectivity, and transduction efficiency of AAV products.^112^

AF4 is less extensively used for AAV characterization but can serve as an alternative or orthogonal method for separating monomeric, dimeric, and oligomeric AAVs and quantifying large aggregates (from virus fragments less than 10 nm in radius to large aggregates above 100 nm in radius).^61,111^ AF4 should represent the first choice when extensive and severe aggregation is expected to occur or to preliminarily characterize a new product, whereas when it has been demonstrated that large aggregates do not form under typical storage conditions, SEC is sufficient and can be more practical for routine aggregation monitoring.^29^

The range of CQAs accessible can be further extended on the basis of the detector configuration. When coupled with MD setups, SEC and AF4 enable true multiattribute quantitation from a single analysis. In this configuration, they can be used not only to investigate aggregation but also to determine total capsid titer and quantify relative capsid content, including estimation of the full-to-total capsid ratio.^110,113,116,117^

### Virus-like Particles

VLPs are noninfectious, self-assembled capsids that lack genetic material. VLPs are primarily used as platforms for vaccine development, both prophylactic and therapeutic. Their ability to present viral antigens in a highly immunogenic form makes them a powerful tool in immunotherapy.^118,119^ The growing interest in VLPs within the pharmaceutical and biotechnology industries has followed the success of blockbuster VLP-based vaccines against hepatitis B and human papillomavirus.^120^

While the analytical characterization of VLPs shares several similarities with that of rAAVs, important differences arise from their broader size range and higher structural heterogeneity. VLP size is strongly dependent on viral origin and is typically larger than that of AAVs, ranging from approximately 30–60 nm for nonenveloped (naked) VLPs up to 80–200 nm for enveloped VLPs (eVLPs), which possess an outer lipid membrane. In addition, due to their self-assembly process, VLP preparations are intrinsically heterogeneous and may contain capsid protein monomers, multimeric or oligomeric assembly intermediates, fully assembled VLPs, and higher-order aggregates.^121^

Both SEC^122–124^ and AF4 ^125–127^ have been widely employed for the analysis and purification of naked VLPs. AF4 is an established and powerful technique for separating viruses and VLPs from other sample components, and AF4-MALS is extensively used for quality assessment, including the determination of particle size, size distribution, and the detection of fragments and aggregates in purified VLP preparations. Furthermore, AF4–MALS enables comparison of VLP quality across different expression hosts and purification strategies.^116^

SEC provides high selectivity for the separation of low-MW impurities (unassembled proteins or smaller oligomers) from assembled VLPs. However, SEC typically elutes intact VLPs in the void fraction while retaining lower-MW species, thus losing information on potential aggregates. Therefore, SEC analysis of VLPs requires columns with suitable pore size (ultralarge pores ranging from 1000 Å to over 2000 Å) to prevent shear degradation and maintain high resolution, and highly hydrophilic surfaces to eliminate secondary interactions.

The development of a 2D-LC approach, which allows the sequential use of SEC columns with different pore sizes, has been reported.^128^ This SEC-SEC-UV-MALS-dRI method enabled the separation of disassembled, intact, and aggregated VLP species within a single run. In the reported setup (Figure 7), the first dimension employed an SEC column with a pore size of 250 Å, and fractions eluting in the void volume were directed to the second dimension using a heart-cutting strategy. The second dimension used a column with a larger pore size (2000 Å), which is suitable for the retention of intact VLPs and the separation of aggregates.^128^

**Figure 7:**
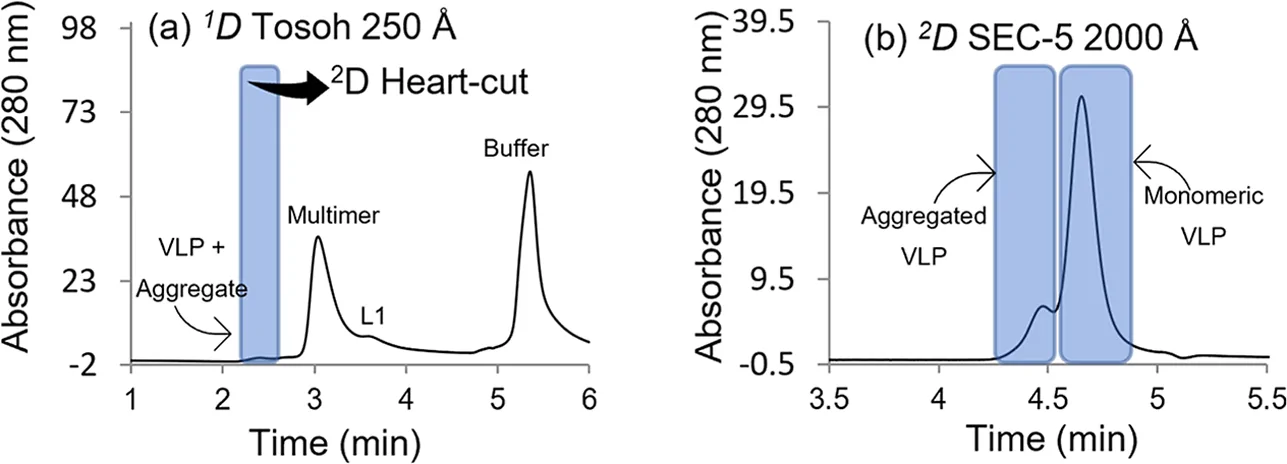
Separation of VLP and aggregates using two SEC columns in 2D-LC mode. (a) First dimension separation using a TSKgel Super SW3000 (4.6 mm × 150 mm, 5 μm). Separation was performed under isocratic conditions at 0.40 mL/min at room temperature. (b) Second dimension separation of the single heart-cut using SEC-5 2000 Å (4.6 × 300 mm, 5 μm). Reproduced with permission from ref 128. Copyright © 2024 American Chemical Society.

Recently, Parikh et al. coupled SEC with high-throughput charge detection-MS (CD-MS), enabling the separation and mass measurements of different species in bacteriophage Qβ and hepatitis B VLP samples. CD-MS is an emerging single-particle MS technique in which both the *m*/*z* and the charge of individual ions are measured simultaneously, enabling direct determination of their mass. The use of narrow-bore SEC columns allowed the direct coupling of SEC to CD-MS, which is particularly advantageous for the analysis of very large and heterogeneous biomolecular assemblies. The study highlighted the complementarity between SEC-CD-MS and SEC-MALS, as the combination of high mass accuracy with biophysical characterization enabled a more detailed analysis of VLP populations and facilitated the discrimination between stable solution aggregates, weakly associated species, and electrospray artifacts.^129^

eVLPs introduce additional analytical complexity because of the presence of an outer lipid bilayer, which increases particle size and structural flexibility and contributes to greater compositional and morphological heterogeneity.

SEC has been used to purify and quantify eVLPs using columns with porosity ranging from 250 to 1000 Å and eluting them in the void fraction. This approach cannot discriminate between particle structural heterogeneity or detect aggregates.^130^ Recently, SEC-MALS using a TSKgel G5000PWxl column has demonstrated the feasibility of absolute size and particle concentration measurements of eVLPs.^131^ However, the method relied on simplifying assumptions regarding particle shape and homogeneity.

AF4–MALS has shown promising capabilities for the characterization and quantification of eVLPs in both purified and unpurified samples. Although Bacher and co-workers reported partial coelution of eVLPs with DNA and chromatin, which may limit quantitative interpretation in complex process-derived samples, the LS90° peak area obtained by AF4–MALS showed a strong correlation with NTA-based particle counts, demonstrating its utility as a reliable tool for both quality control and in-process monitoring.^132^

While the literature on the use of SEC and AF4 for naked VLP purification, characterization, and quality assessment, particularly with respect to aggregation, is well established, studies applying these techniques to eVLPs remain scarce and fragmented. This disparity reflects the intrinsic structural complexity and mechanical fragility of eVLPs, which make them prone to secondary interactions and shear-induced perturbations and highlight the need for further optimization to ensure reliable size characterization.^132^

### Polymeric NPs

In the field of drug delivery, polymeric NPs (PNPs) are gaining increasing attention due to their structural diversity and versatility, as well as their ability to encapsulate a wide range of therapeutic agents and to be surface-functionalized for targeted delivery.^16,133^ Pharmaceutically relevant PNPs typically fall within the range 10–300 nm and are composed of biodegradable or biocompatible polymers.^133,134^

PNPs encompass structurally diverse systems whose architectural features directly influence their behavior under size-based separation techniques. Despite their structural diversity, they can be evaluated through a common set of parameters, including particle size, PSD, stability, and drug release kinetics. PNPs are discussed here as molecularly defined polymeric nanocarriers, such as polymer–drug conjugates (PDCs) and dendrimers, micelles, and solid PNPs. Single-chain NPs (SCNPs), obtained by intramolecular folding of individual polymer chains, have also been used as model polymer nano-objects to compare MD-SEC and AF4 approaches. A direct comparison demonstrated how the structural characterization of compact polymer nanostructures can strongly depend on the separation principle and the detection system employed, as AF4 provided a more consistent description of polymer compaction, whereas SEC may be limited by the similar hydrodynamic volumes of linear and collapsed species and potential interactions with the stationary phase.^135^

### Molecularly Defined Polymeric Nanocarriers: PDCs and Dendrimers

PDCs and dendrimers behave as discrete macromolecular entities whose characterization is more closely aligned with polymer analytical methodologies than with typical NP analysis. PDCs consist of therapeutic agents covalently attached to polymer chains, representing a versatile strategy to enhance drug solubility, modulate pharmacokinetics, and achieve controlled release.^136^ Over the past decade, numerous PDC-based formulations have entered the market, and several others are in clinical trials.^137^ A related class of covalent polymer nanocarriers is represented by dendrimers, highly branched macromolecules with a well-defined architecture and multiple terminal functional groups that can be exploited for drug conjugation or surface functionalization. In both systems, the increasing conjugate variability and synthetic complexity lead to significant heterogeneity in drug loading and MW distribution, making comprehensive physicochemical characterization essential to ensure product quality.^137^

The distinctive molecular nature and size of these macromolecular drug carriers, which differentiate them from the other NPh systems discussed above, make SEC, including gel permeation chromatography (GPC) in organic solvents, particularly suitable for characterizing their formation and monitoring conjugation reactions. SEC/GPC has been widely used to monitor PDC and dendrimer synthesis, assess purity, and detect the presence of lower- or higher-MW species arising from incomplete growth or degradation.^138–140^ Depending on polymer solubility, analyses are typically performed using *N*,*N*-dimethylacetamide (DMAc) or dimethylformamide (DMF) as eluents for hydrophobic polymers,^139,141–143^ and aqueous mobile phases for hydrophilic systems.^141^ MW determination is generally carried out using a UV and/or dRI detector and calibration against polymer standards.^141^ However, for highly branched architectures such as dendrimers, calibration with linear polymer standards may lead to deviations due to their more compact hydrodynamic structure;^144^ therefore, absolute MW is more reliably determined using MALS.^145^

A peculiar class of PDCs is represented by protein–polymer conjugates, which combine the unique properties of both proteins and synthetic polymers and are increasingly relevant for biomedical applications.^146^ In this context, SEC is commonly coupled with MALS to determine absolute MW and characterize conjugation efficiency. Recently, Kapil et al. applied an SEC-MALS method to characterize protein-branched polymer bioconjugates, monitoring the conjugation process over time and assessing absolute MW.^146^

Because of their discrete macromolecular nature, PDCs and dendrimers are primarily characterized by SEC, whereas AF4 is less frequently applied. AF4 becomes more relevant when association phenomena, labile complexes, or higher-order nanoscale assemblies need to be investigated.^25,147^

### Polymeric Micelles

Polymeric micelles (PMs) are the preferred carrier system for hydrophobic drugs and represent a distinct class of NPhs formed through the spontaneous self-assembly of amphiphilic block copolymers in aqueous environments.^148–150^ PMs are dynamic supramolecular constructs with diameters of 20–100 nm whose structural integrity depends on concentration, solvent conditions, and temperature. This dynamic nature directly impacts their analytical characterization, as size, aggregation number, and stability may vary under separation conditions.^150^ Cross-linking strategies are frequently introduced in PMs to prevent premature drug release by stabilizing the micellar core or shell, thereby enhancing structural robustness.^151^ These reinforced nanocarriers are generally expected to better withstand separation processes.^148^ In contrast, non-cross-linked PMs remain intrinsically dynamic and susceptible to dilution-induced disassembly. While AF4 is expected to be suitable for preserving micellar integrity, literature evidence also suggests that SEC can maintain the structural integrity of non-cross-linked micelles, provided that concentration-dependent dissociation effects are carefully controlled.^152–154^ The ability of size-based techniques to preserve micellar integrity has been strategically exploited in SEC/GPC to separate intact micelles from constituent polymers^154^ or released drug,^152^ enabling the evaluation of EE and release behavior. In contrast, AF4 appears particularly well-suited for investigating micellar stability under biologically relevant conditions, including complex media such as plasma, where changes in size distribution and polydispersity can be monitored.^149,150,155,156^ When coupled to MD systems, AF4 further enables the simultaneous assessment of micellar integrity and drug content within a single run, allowing drug release associated with carrier destabilization to be distinguished from diffusion-driven release.^149,150,156^

Both SEC and AF4 have been applied for cross-linked and non-cross-linked micelle characterization, albeit with different implications for structural preservation and data interpretation. While SEC can detect the formation of high-MW species and aggregates, its accuracy may be affected by adsorption phenomena.^154^ In contrast, AF4 provides a more reliable assessment of aggregation state and size distribution.^155,157^

### Solid PNPs

Solid PNPs consist of dense polymer matrices in which the therapeutic payload is either encapsulated or dispersed within the solid core. In contrast to dynamic self-assembled systems, these nanocarriers behave as discrete colloidal particles with a defined size distribution and a higher degree of structural stability. Their characterization therefore primarily relies on an accurate determination of particle size, polydispersity, and aggregation state.

In this context, AF4 has been largely applied to the characterization of solid PNPs,^16,31^ including polylactic acid (PLA)-PEG,^158^ poly­(lactic-*co*-glycolic acid) (PLGA),^159^ and poly­(l-lactic acid) (PLLA)^159^ NPs, even under biologically relevant conditions. Once again, the power of AF4 emerges in its MD setups, which enable the simultaneous determination of several critical NP attributes, including PSD, shape, and drug loading.^25^ AF4 was applied to the determination of the rates and mechanisms of release of a fluorescent antibiotic, enrofloxacin, from PLGA-NPs in buffers and simulated biological media. The Eclipse AF4 short channel was configured with a wide spacer with a spacer height of 350 μm and a 10 kDa MWCO RC membrane. The spacer height determines the thickness of the separation channel and has a major influence on separation performance, sample recovery, and analysis time.^28^ AF4 enables the in situ separation and removal of nonencapsulated drug, while the combined use of online FLD and UV detection allows selective quantification of the encapsulated drug and the PLGA-NP fraction, respectively. This integrated approach thus permits the determination of drug release directly during the AF4 analysis in a single run with an analysis time of 100 min^160^

In 2024, Klein et al. reported a robust AF4-MD method for the characterization of a novel HIV nanovaccine (average diameter 160 nm) based on electrostatic assembly of antigen with chitosan and dextran sulfate. The approach enables, in a single analysis, quantification of free antigen (UV absorption) together with the determination of *R*_g_, size distribution, and size-dependent shape (MALS). The authors performed the AF4 separation with three different membranes (an RC membrane with a 5 kDa cutoff, another RC membrane with a 10 kDa cutoff, and a 5 kDa PES membrane) and three different mobile phases (ultrapure water, 0.1 M NaCl, and 0.1% (w/v) SDS). The best results were obtained with an RC membrane. Regarding the eluent composition, water promoted particle adsorption, and buffer solution induced particle disruption, while 0.1% (w/v) sodium dodecyl sulfate (SDS) was found to ensure high recovery and minimize matrix effects. In this case, despite its low MW (<10 kDa), the free antigen remained associated through electrostatic interactions and eluted with satisfactory recovery.^161^ Despite this specific case, in general, AF4 allows determination of released drug and EE only when the drug is a high-MW molecule (e.g., a protein), as small molecules below the membrane MWCO permeate the membrane and are removed by the cross-flow.^162^

In contrast, the current contribution of SEC in the analysis of solid PNPs is mainly confined to its historical role in the characterization of the polymeric materials forming the NP matrix, in terms of MW, dispersity, and stability.^163^ SEC use remains limited due to potential non–size-based interactions with the stationary phase and shear stress effects.^164^ Indeed, the recent advances in SEC technology that have significantly expanded its use for other NP classes, such as LNPs or viral vectors, have not yet been widely translated to PNP systems. This limitation is related to the heterogeneous and often highly polydisperse nature of PNPs, as well as to the presence of hydrophobic polymer matrices and stabilizing surfactants, which promote secondary interactions with the stationary phase.

### Inorganic NPs

INPs represent an important class of nanomaterials in pharmaceutical and biomedical applications, where they have been explored as drug delivery systems, imaging agents, and theragnostic platforms.^165,166^ INPs used in NPh research comprise rigid, high-density nanostructures, including metal-, metal oxide-, and silica-based systems as well as quantum dots (QDs).^167^ In particular, noble metal NPs have attracted considerable attention due to their outstanding optical, catalytic, and antimicrobial properties, along with their potential for targeted drug delivery.^168^ Despite their compositional diversity, these materials share common structural characteristics such as relatively high density, limited deformability, and tunable surface chemistry. In pharmaceutical applications, INPs typically range from a few nanometers to about 100 nm, depending on the core material and surface functionalization.^169^ Their rigid structure, defined by synthetic control rather than self-assembly processes, makes them particularly amenable to analysis by both SEC and AF4.

SEC has been effectively applied to the analysis of INPs. SEC coupled with UV–vis detection has been used to characterize the size distributions and separate multimodal mixtures of gold, silver, and silica NPs using calibration curves constructed with gold NP (AuNP) standards (5–80 nm).^170^ The stationary phase used consisted of particles with a diameter of 10 μm and an average pore size of 100 nm. When the amount of sample injected into the SEC column was increased 10-fold, the resolution was still good, suggesting that the technique can likely handle larger sample quantities without losing its ability to separate components effectively. Citrate-stabilized gold nanorods (GNRs) were separated according to their hydrodynamic size using a silica-based SEC column (pore size of 1000 Å), while the UV–vis diode array detector (DAD) enabled reconstruction of their length and diameter distributions by exploiting the geometry-dependent plasmonic response of the nanorods.^171^ In a recent paper, a gravity-flow SEC technique was used at semipreparative and preparative scale for the separation of Au nanomolecules protected by different ligands (phenylethanethiolate, thiophenolate, 4-*tert*-butylbenzene thiolate, and *tert*-butyl thiolate). The separation of NPs of different sizes protected with different types of ligands demonstrates that SEC can be used for isolation of highly monodisperse nanomolecules, regardless of the ligand type.^172^

AF4 is a well-established methodology to analyze and characterize a wide range of INPs.^33^ Recently, AF4 was applied to compare the elution behavior of AuNPs with three different morphologies [gold nanospheres (GNSs), gold nanotriangles (GNTs), and GNRs] and to provide a comprehensive understanding of their separation mechanism in AF4. Although the GNSs and GNRs had similar dimensions based on TEM measurements, the GNRs eluted significantly earlier in AF4 than did the GNSs. This suggests that particle shape, not just size, influences transport and separation behavior in the AF4 channel. The study demonstrates that AF4 shows strong potential as a specialized technique for separating and analyzing NPs based on shape as well as size.^173^

For the analysis of metal-containing NPs, AF4 and SEC are frequently coupled with ICP–MS, which provides high sensitivity and element-specific detection capabilities. ICP–MS can be operated in both conventional mode, providing bulk elemental composition of size-separated fractions, and single-particle mode (spICP-MS), which enables determination of particle number concentration and elemental mass at the individual particle level. Conventional ICP–MS analyzers (e.g., quadrupole or sector field) detect isotopes sequentially and therefore require prior knowledge of the sample composition to avoid information loss. More recently, the coupling of AF4 with ICP time-of-flight MS (ICP-TOFMS) has been proposed, enabling simultaneous detection of all isotopes and facilitating untargeted elemental analysis of AF4-separated particle populations.^174^ AF4-ICP-MS has been applied to the size-based separation and quantification of silica NPs, enabling size-resolved analysis and determination of NP concentrations in complex matrices and biological samples.^175^ Recently, AF4–ICP–MS has also been applied to the separation and quantification of ionic and nanoparticulate species of the same element. When applied to Resovist, a nanomedicine composed of carboxydextran-coated iron oxide NPs, the method enabled the detection of as little as 0.022% of Fe in ionic form while simultaneously assessing particle size and aggregation.^176^

AuNPs have been widely investigated due to their tunable surface properties and ease of functionalization with therapeutic agents and biomolecules, enabling applications in drug delivery, bioimaging, and diagnostics.^177^ AuNPs are typically capped with stabilizing agents (e.g., citrate or PEG), which can be further functionalized or modified through interactions with endogenous or therapeutic proteins. In this context, AF4 has been proposed as a platform for multiparameter characterization of NP-protein bioconjugates, enabling the evaluation of conjugation efficiency and stability.^178,179^ AF4 enabled the resolution of different components in the system, including the unmodified AuNPs, the free protein, and the NP–protein conjugates, while preserving their native structure. A comparable AF4-based approach has also been applied to oligonucleotide-functionalized gold nanostructures. Here, ICP–MS/MS has been integrated to enable interference-free detection of P and S, allowing the determination of oligonucleotide surface coverage and further demonstrating the versatility of AF4 for the characterization of NP-biomolecule conjugates.^180^

A similar capability has also been demonstrated using SEC column cartridges (qEVoriginal, iZon Science, 70 nm pore size, void volume = 3 mL). For citrate-capped AuNPs (∼13 nm) functionalized with different proteins, SEC enabled the separation of NP-protein conjugates from both free proteins and unmodified NPs, providing a straightforward method to confirm successful surface functionalization.^181^

QDs, semiconductor nanocrystals widely used as fluorescent probes for bioimaging and bioanalytical applications, have also been investigated by AF4 and SEC. AF4 enabled the size-resolved study of QD–protein interactions in biological media as well as the separation and characterization of QD-antibody bioconjugates.^182,183^ In one study, SEC–ICP–MS was employed to monitor Cd^2+^ release from CdSe@ZnS QDs (∼4.6 nm) by separating intact NPs from dissolved ions. The method addressed the strong adsorption of both species onto the SEC column using a mobile phase containing EDTA to complex dissolved ions and SDS to stabilize the NPs and minimize nonspecific interactions with the column.^76^

Finally, the AF4-MD platform has also been applied to characterize AuNPs with different surface properties and their conjugates^184^ and to investigate drug release behavior from INP-based delivery systems. A robust yet flexible method was first developed that could be applied to different AuNPs and conjugating molecules. The separations were performed using an RC membrane with a 10 kDa MW cutoff in a 300 mm channel with a 350 μm-thick spacer. Crossflows and mobile phase composition were optimized to improve sample recovery. In 2025, Marassi et al. reported an AF4-DAD-MALS platform to characterize a model NPh system based on bovine serum albumin-coated AuNPs loaded with curcumin. An exponential decay model was applied to the crossflow rate to enhance separation efficiency. The MD-AF4 setup provided size-resolved information on NP structure, drug loading, and stability, and allowed real-time monitoring of drug release under physiologically relevant conditions using the absorbance ratio *A*_401_/*A*_530_ as an indicator of curcumin release. Overall, the study highlights the potential of AF4 as a powerful analytical tool for the simultaneous evaluation of AuNP integrity, drug loading, and release behavior.^177^

Overall, the literature suggests that the structural rigidity and limited deformability of INPs make them generally well-suited for separation by both SEC and AF4. Within this framework, SEC offers a robust, simple, and accessible platform for routine characterization of relatively monodisperse INPs. However, AF4 offers clear advantages for the advanced characterization of complex, heterogeneous, and biologically functionalized NPs. For both techniques, the analytical information obtained ultimately depends on the detection system coupled with the separative method. Coupling either technique with MALS and DLS provides critical, complementary information regarding the PSD and morphological characteristics. Similarly, the coupling with ICP–MS or multidetection platforms enables robust size-based resolution alongside precise elemental identification and quantification, as well as the assessment of drug loading and drug release behavior.

Figure 8 summarizes the main CQAs identified from the application of AF4 and SEC in the characterization of the different classes of NPhs, based on the literature published since 2020. The list of CQAs is not exhaustive and reflects the most relevant attributes, depending on the specific application. The indicative publication counts reporting the use of AF4 and SEC within the same time frame are also included for each class.

**Figure 8:**
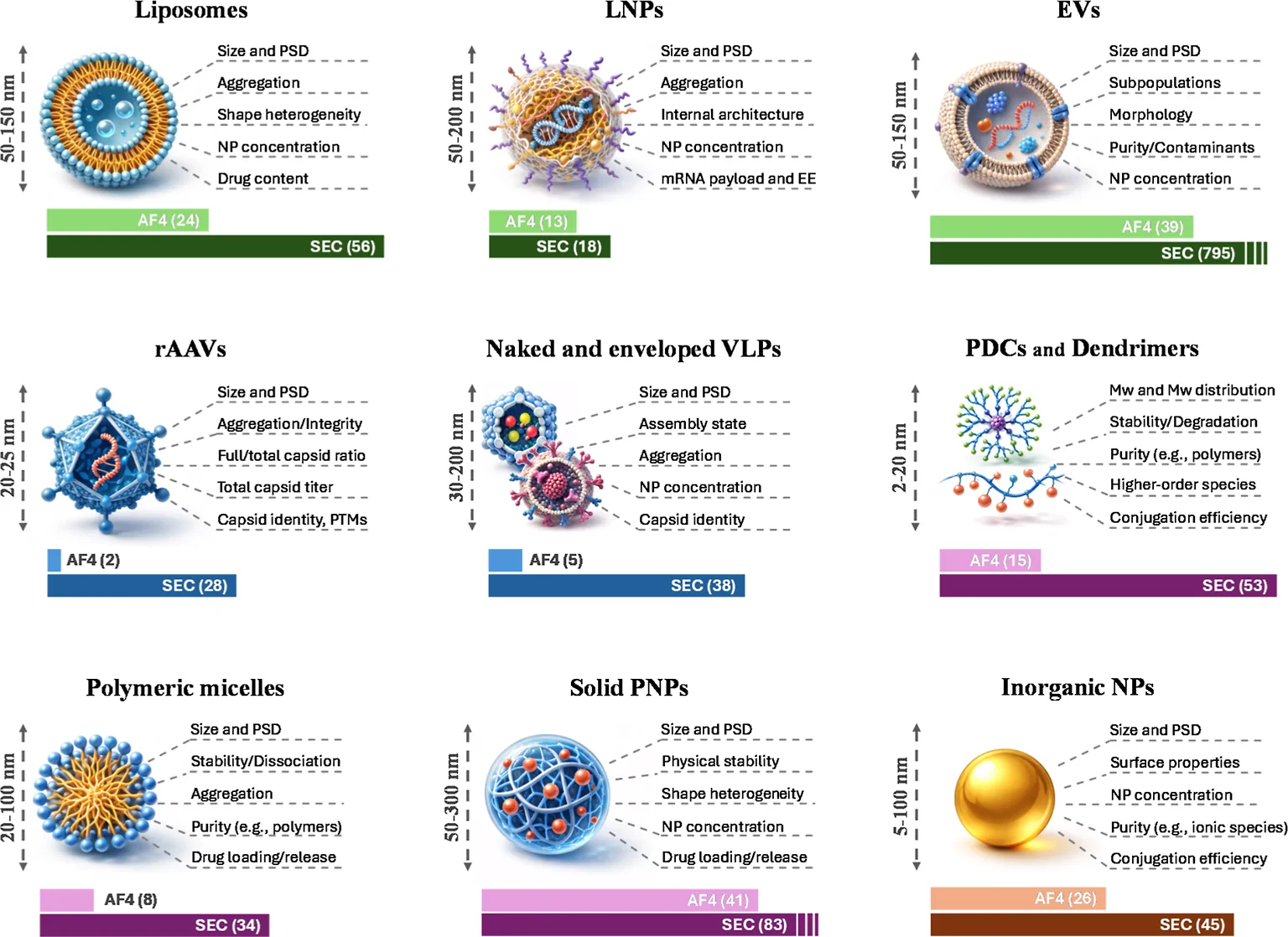
Overview of the main CQAs identified from the application of AF4 and SEC in the characterization of different classes of NPhs, based on literature since 2020. Indicative publication counts for each technique within the same time frame are also reported and were determined by searching PubMed using the combination “nanoparticle class” + “technique” in March 2026. The high number of publications reporting SEC in the analysis of EVs and solid PNPs reflects its widespread use as an isolation method and for characterization of polymeric materials, respectively. Color coding: lipid-based systems (green), virus-based systems (blue), inorganic nanoparticles (orange), and polymer-based systems (purple).

## Regulatory Considerations

The development and diffusion of NPhs have been accompanied by the need to establish and implement an appropriate regulatory framework. Moreover, the expiration of patents for several NPhs, together with the rise of nanosimilars, has intensified the need for clear regulatory guidance and rigorous analytical standards to demonstrate nanosimilarity.^185^ This growing attention over the past decade has led to the publication of several guidance documents and reflection papers addressing the specific challenges associated with nanoscale drug products. These documents aim to support the development, approval, and commercialization of drug products based on nanomaterials and ensure their safety, efficacy, and manufacturing consistency. Guidelines and reflection papers have identified physicochemical attributes of NPhs that should be characterized to support the marketing authorization of products developed as innovative products or as follow-on products referencing an innovator. These attributes include mean size and size distribution, morphology, and physical stability (e.g., aggregation). However, general regulatory documents consistently emphasize that the selection of CQAs should be adapted to the specific types of nanomedicines under development.

Within the European regulatory framework, the EU-IN Horizon Scanning Report on nanotechnology-based medicinal products for human use,^8^ released by EMA in 2025, highlights the need for comprehensive physicochemical characterization to adequately describe nanoscale systems. Additional product-specific guidance has been developed for particular classes of nanomedicines, initially addressing liposomal formulations^186^ and subsequently covering other nanosystems, including iron-based and nanocrystal-based products,^187^ whose patents are approaching expiration. Similarly, the FDA has issued guidance documents addressing nanotechnology in drug products, including the guidance for industry: drug products, including biological products, that contain nanomaterials^188^ and the guidance liposome drug products chemistry, manufacturing, and controls; human pharmacokinetics and bioavailability; and labeling documentation.^189^ All of these documents emphasize the importance of detailed characterization of particle size and size distribution as key quality attributes influencing pharmacokinetics, biodistribution, and safety.

However, at present, only a limited number of internationally standardized test methods are available for the size characterization of nanomedicines, and most existing methods may not be sufficient to fully describe their CQAs. Particle size determination in routine quality control is still largely based on DLS applied as an ensemble method, providing limited information on heterogeneous or polydisperse systems.^97^ A significant gap thus remains between the regulatory need for robust and validated characterization methods to support decision-making and the current availability of such methods for complex NPh products.^39^ For this reason, regulatory frameworks emphasize the importance of applying multiple complementary and orthogonal analytical techniques to accurately measure size distributions and detect subpopulations or aggregates.

In this context, size-based separation techniques play a crucial role. SEC is currently widely established in regulatory submissions for the characterization of proteins and certain NP-based formulations, mainly due to its robustness, high level of standardization, and compatibility with quality control environments.^20,190^ Nevertheless, as highlighted throughout this Review, the applicability of SEC to complex NP systems is still evolving. As the regulatory expectations for the physicochemical characterization of NPhs continue to expand, it is likely that the role of SEC within the analytical toolbox will also evolve over the coming years.

The application of AF4 to NPhs has increased substantially in recent years, attracting growing interest within the regulatory and standardization landscapes. In 2018, the first international technical specification for the analysis of nano-objects using AF4 (ISO/TS 21362)^191^ was published, representing an important step toward method harmonization. This specification was recently updated and evolved into the international standard ISO 21362 in February 2026.^192^ Initiatives from collaborative networks, such as EUNCL and NCI-NCL, have contributed to the development of such standardized protocols.^37,193^ Although AF4 is not yet as widely standardized as SEC in regulated environments, the increasing availability of standardized procedures and interlaboratory initiatives suggests that its role in NPh quality control will continue to expand.

Moreover, both techniques are compatible with MD platforms, enabling the simultaneous assessment of multiple physicochemical attributes. This capability aligns well with the increasing regulatory emphasis on multiattribute and orthogonal analytical strategies, further enhancing their future roles in the comprehensive characterization of complex NPh products.

## Conclusions

This review synthesizes recent advances in AF4 and SEC for the characterization of NPh size and size distribution.

As with any analytical approach, the choice between SEC and AF4 involves balancing their inherent advantages and limitations against the type of information required and the associated resources needed for method development and analysis. AF4 offers clear advantages over SEC in certain aspects of NPh characterization, particularly due to the absence of a stationary phase that could interact with or damage NPs. This feature can support high-fidelity assessment of particle sizing, recovery, and the detection of fragile or interaction-prone species, making AF4 especially valuable for detailed characterization, stability studies, and regulatory assessment. However, AF4 is not entirely artifact-free, and recovery, membrane interactions, sample loading, and carrier conditions should be carefully controlled to preserve the original state of the sample during analysis. Additionally, despite the advantages mentioned above, AF4 adoption has remained relatively limited, often focused primarily on sizing applications, reflecting practical constraints such as instrument cost, the need for specialized expertise, and the scarcity of robust, broadly applicable protocols. Meanwhile, SEC remains the most well-established and extensively characterized size-based chromatographic method in the pharmaceutical field. Ultrawide pore columns and novel stationary phases that reduce analyte–stationary phase interactions are expanding SEC applicability to a broader NP size range. SEC offers practical advantages such as faster analysis, operational robustness, high reproducibility, and suitability for automated high-throughput workflows, making it particularly attractive for quality control. While many experts in the field remain skeptical about the role that SEC is expected to play in NPh analysis, the sustained efforts of manufacturers to advance surface chemistry of packing materials, pore architecture, and column hardware clearly reflect an existing analytical need and are likely to foster the development of more efficient SEC columns tailored for NPh characterization.

Overall, SEC and AF4 should be viewed as complementary, rather than competing, techniques. SEC provides operational reliability and throughput, while AF4 excels in versatility and investigation of aggregation. Their combined application enables comprehensive characterization of NPhs, supports regulatory requirements, and allows for informed decision-making throughout NP development, quality control, and stability assessment. As both techniques continue to evolve, leveraging their respective strengths will further expand the analytical toolkit available for the next generation of NPhs.

## Abbreviations

NPhs: nanopharmaceuticals
API: active pharmaceutical ingredient
NP: nanoparticle
CQA: critical quality attribute
EE: encapsulation efficiency
DLS: dynamic light scattering
NTA: nanoparticle tracking analysis
TDA: Taylor dispersion analysis
AF4: asymmetric flow field-flow fractionation
SEC: size exclusion chromatography
HDC: hydrodynamic chromatography
ELS: electrophoretic light scattering
CE: capillary electrophoresis
cryo-TEM: cryogenic transmission electron microscopy
LC: liquid chromatography
MS: mass spectrometry
EMA: European Medicines Agency
FDA: food and drug administration
UV: ultraviolet
dRI: differential refractive index detector
FLD: fluorescence detector
MALS: multi-angle light scattering
MW: molecular weight
Rg: radius of gyration
DLS: dynamic light scattering
Rh: hydrodynamic radius
ESI: electrospray ionization
ICP-MS: inductively coupled plasma mass spectrometry
PSD: particle size distribution
MD: multi-detector
LNPs: lipid nanoparticles
AAV: adeno-associated virus
VLPs: virus-like particles
PDI: polydispersity index
FI: frit-inlet
RC: regenerated cellulose
PES: polyethersulfone
h-HST: hydrophilic hybrid surface technology
HF5: hollow-fiber flow FFF
EUNCL: European Nanomedicine Characterization Laboratory
NCI-NCL: National Cancer Institute Nanotechnology Characterization Laboratory
CDER: Center for Drug Evaluation and Research
SLNs: solid lipid nanoparticles
NLCs: nanostructured lipid carriers
LNCs: lipid nanocapsules
EVs: extracellular vesicles
IV: inline viscometer
PEG: polyethylene glycol
SAXS: small-angle X-ray scattering
LVSS: large-volume sample stacking
dUC: differential ultracentrifugation
rAAV: recombinant adeno-associated virus
PTMs: post-translational modifications
CD-MS: charge detection mass spectrometry
eVLPs: enveloped virus-like particles
PNPs: polymeric nanoparticles
SCNPs: single-chain nanoparticles
PDCs: polymer-drug conjugates
GPC: gel permeation chromatography
DMAc: *N*,*N*-dimethylacetamide
DMF: dimethylformamide
PMs: polymeric micelles
SDS: sodium dodecyl sulfate
INPs: inorganic nanoparticles
QDs: quantum dots
AuNPs: gold nanoparticles
DAD: diode array detector
GNS: gold nanospheres
GNT: gold nanotriangles
GNR: gold nanorods
spICP-MS: single particle ICP-MS
ICP-TOFMS: inductively coupled plasma time-of-flight mass spectrometry
PLA: polylactic acid
PLGA: poly­(lactic-*co*-glycolic acid)
PLLA: poly­(l-lactic acid).
